# PRAME Augments K63‐Linked Ubiquitination and Stabilization of METTL3 to Promote Lung Adenocarcinoma Metastasis

**DOI:** 10.1002/mco2.71066

**Published:** 2026-10-19

**Authors:** Xia Yang, Xuemei Xu, Yaxuan Zhang, Jiamei Guo, Zhiyu Ye, Qiaorui Bai, Xiuqiao Yang, Shujun Liang, Xiang Dong, Xin Zhang, Jiyong Gu, Shuqin Dai, Hongchun Du, Meiwan Chen, Tianyu Tao, Hongyu Guan, Jueheng Wu, Han Tian, Junchao Cai, Mengfeng Li, Xu Li, Yi Yang

**Affiliations:** ^1^ Department of Laboratory Medicine and Institute of Precise Medicine, The First Affiliated Hospital Sun Yat‐sen University Guangzhou Guangdong China; ^2^ Cancer Institute, School of Basic Medical Sciences Southern Medical University Guangzhou Guangdong China; ^3^ Zhongshan School of Medicine Sun Yat‐Sen University Guangzhou Guangdong China; ^4^ Clinical Experimental Center, Jiangmen Engineering Technology Research Center of Clinical Biobank and Translational Research Jiangmen Central Hospital Jiangmen Guangdong China; ^5^ Department of Neurosurgery Jiangmen Central Hospital Jiangmen Guangdong China; ^6^ Sun Yat‐sen University Cancer Center Guangzhou Guangdong China; ^7^ Department of Pulmonary and Critical Care Medicine, The First Affiliated Hospital Sun Yat‐sen University Guangzhou Guangdong China; ^8^ State Key Laboratory of Mechanism and Quality of Chinese Medicine, Institute of Chinese Medical Sciences University of Macau Macau SAR China; ^9^ Department of Endocrinology and Diabetes Center, The First Affiliated Hospital Sun Yat‐sen University Guangzhou China; ^10^ State Key Laboratory of Multi‐Organ Injury Prevention and Treatment Southern Medical University Guangzhou Guangdong China

**Keywords:** K63‐linked ubiquitination, LUAD, metastasis, METTL3, PRAME

## Abstract

Metastasis is the primary cause of mortality in cancer including lung adenocarcinoma (LUAD), yet the underlying mechanisms remain incompletely understood. Preferentially expressed antigen of melanoma (PRAME), a cancer‐testis antigen, displays context‐dependent functions across malignancies, but its biological role in LUAD metastasis has not been clearly defined. Here, we demonstrate that the significantly upregulated PRAME in metastatic LUAD directly interacts with the m^6^A methyltransferase METTL3 and promotes its K63‐linked non‐proteolytic ubiquitination. This modification occurs at lysine 235 of METTL3, increases its protein stability and facilitates METTL3 interaction with METTL14 and WTAP. Consequently, m^6^A modification of *TGFB2* mRNA and activation of the TGF‐β signaling pathway are enhanced, which is essential for PRAME‐driven pro‐metastatic effects both in vitro and in vivo. Clinically, PRAME expression positively correlates with METTL3 protein abundance and TGF‐β pathway activation, and is robustly associated with poor clinical outcomes. Taken together, our findings identify K63‐linked ubiquitination as a previously unrecognized post‐translational modification of METTL3 and underscore the importance of the PRAME/METTL3/TGF‐β axis in LUAD metastasis, providing mechanistic insights and potential therapeutic targets for this malignancy.

## Graphical abstract

PRAME promotes lung adenocarcinoma metastasis by inducing K63‐linked ubiquitination of METTL3 at lysine 235, thereby enhancing METTL3 stability and m^6^A methyltransferase complex assembly. Elevated METTL3 activity increases m^6^A modification of *TGFB2* mRNA, leading to activation of TGF‐β signaling and metastatic progression of lung adenocarcinoma (LUAD).

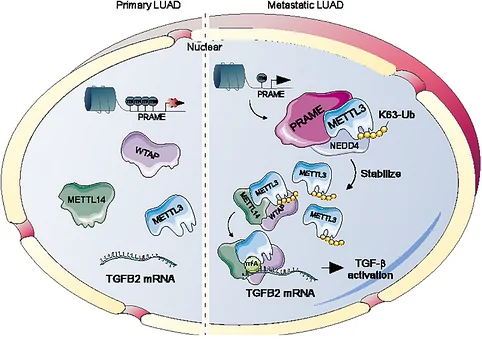

## 1 Introduction

Lung adenocarcinoma (LUAD) represents the predominant histological subtype of lung cancer and remains a major contributor to cancer‐related deaths worldwide, with tumor dissemination being a key determinant of its unfavorable clinical outcomes [1]. Due to the generally imperceptible symptoms in early LUAD, over 50% of patients present with intrapulmonary or distant metastases at the time of initial diagnosis, resulting in an overall 5‐year survival rate of only approximately 20% for LUAD patients, and less than 5% for those with metastatic LUAD [2]. Despite substantial progress in therapeutic strategies, the clinical outcomes of patients with metastatic LUAD remain poor, with a median survival of only approximately 5 months and more than half of patients succumbing to the disease within one year [3, 4]. Metastatic LUAD patients frequently respond poorly to current therapies and face high rates of relapse, underscoring the urgent need to identify new and effective therapeutic targets.

Preferentially expressed antigen of melanoma (PRAME) was initially identified as a tumor‐associated antigen in human melanoma. Because of its widespread expression across many tumor types but absence or very low expression in most normal tissues except testis [5, 6], PRAME is defined as a cancer‐testis antigen (CTA) and is considered a promising candidate target for anticancer immunotherapies [7]. In fact, immunotherapy targeting PRAME has been shown to induce robust immune responses in healthy volunteers and patients with leukemia or melanoma, and its potential application as an anticancer vaccine in both hematologic malignancies and solid tumors including lung cancer, has been clinically evaluated [8, 9, 10, 11]. Interestingly, in contrast to the remarkable efficacy of PRAME immunotherapeutic approach in eliciting potent CD8^+^ T‐cell response in leukemia, it fails to do so in lung cancer, but rather provokes a humoral response [10, 12], indicating that the immune response induced by PRAME can be tumor type‐ and context‐dependent. Moreover, high expression of PRAME does not indicate favorable prognosis of LUAD patients [13, 14], suggesting that it might confer a selective advantage to the tumor, which outweighs its potential value as an immunotherapy target in this malignancy [5]. By and large, the biological role of PRAME in LUAD is still unclear, as some studies report its oncogenic and pro‐metastatic functions [15, 16], while others suggest the opposite [17]. Particularly, how PRAME contributes to LUAD metastasis remains unclarified.

Tumor metastasis is a complex biological cascade that begins with the escape of malignant cells from the primary tumor and culminates in their dissemination to distant organs, and tumor cells require quick switches of diverse phenotypes that confer metastatic competence. It has been well recognized that successive accumulation of both genetic and epigenetic alterations contributes to successful metastasis [18]. Notably, tumor cells are able to drive phenotypic plasticity and clonal evolution required for metastasis through epigenetic changes, thereby dynamically acquiring new molecular features to adapt to the highly variable selective pressures during the multi‐step process [19]. The reversible property and the causal role of epigenetic machinery in metastatic traits have made it an attractive therapeutic target against metastasis [18, 20]. As one of the important epigenetic events, N^6^‐methyladenosine (m^6^A) modification, namely, methylation at the nitrogen‐6 position of adenosine in RNA, has been found to be associated with various pathological conditions, including LUAD metastasis [21]. RNA m^6^A modification is a dynamic process, achieved by the major m^6^A “writer” (methyltransferase) complex comprising METTL3, METTL14, and WTAP [22]. METTL3 is the only catalytically active component of the complex and has been demonstrated to be pro‐metastatic in LUAD [23]. Interestingly, while studies have shown that high METTL3 protein and global m^6^A levels (primarily mediated by METTL3 in LUAD) are associated with advanced LUAD and poorer patient survival [23, 24], no correlation could be detected between *METTL3* mRNA expression and the clinical advancement and outcomes of LUAD in the TCGA dataset [25, 26]. The molecular basis for such a discrepancy between *METTL3* mRNA and protein expression levels in LUAD remains largely unexplored. Dissection of the regulating network of METTL3 mRNA and protein expression might facilitate a better understanding of the biological mechanism for LUAD metastasis.

Our present study reports that the upregulated PRAME facilitates K63‐linked ubiquitination on METTL3 protein mediated by E3 ligase NEDD4, thus promoting METTL3 stability and its affinity with other m^6^A writer components such as METTL14 or WTAP. In addition, by demonstrating *TGFB2* as a METTL3 target, we elucidate the importance of the PRAME/METTL3/TGF‐β axis in LUAD metastasis, potentially providing new insights in the future development of strategies against LUAD metastasis based on the above newly identified molecular mechanisms.

## 2 Results

### 2.1 PRAME is Upregulated in LUAD Metastases and is Related to Poor Prognosis

To investigate the association of *PRAME* with LUAD progression and metastasis, we analyzed public transcriptomic datasets and independent clinical cohorts, and further assessed its relationship with clinical outcomes. Analysis of the TCGA‐LUAD cohort demonstrated that *PRAME* mRNA expression is significantly upregulated in tumor tissues compared with adjacent normal lung tissues (Figure S1A). This observation was further validated across other independent GEO datasets GSE32863 and GSE10072 (Figure S1B), consistently showing that PRAME is aberrantly overexpressed in LUAD, while remaining at low or undetectable levels in normal lung tissues. Beyond the tumor‐versus‐normal comparison, a published scRNA‐seq dataset [27] was also analyzed for differentially expressed genes (DEGs) between tumor cells in metastatic lesions and primary tumors (Figure 1A), and PRAME expression was found to be significantly higher in metastatic tumors (Figures 1A–C and S1C), suggesting an association between elevated *PRAME* expression and the metastatic state of LUAD at the transcriptomic level. In an LUAD cohort of 34 patients (Table S1), PRAME expression in metastatic lesions (29 samples), in comparison with that in corresponding primary tumors (29 samples), was determined by RT‐qPCR and immunohistochemistry (IHC) assays, and a drastic increase in PRAME was revealed (Figure 1D,E). Elevated levels of PRAME were also observed in metastatic tumors of multiple distant target organs including the brain and the bones, following intracardiac injection of the A549 LUAD cells in BALB/c‐nu mice (Figure 1F,G). Furthermore, we detected a dramatic elevation of PRAME expression in two LUAD cell lines with high metastatic capacity (HMC), constructed by isolating and purifying brain metastatic cells and expanding them in culture for three rounds of in vivo selection using a previously described method [28] (Figures 1H and S1D). Together, these clinical, single‐cell, and experimental metastasis models provided complementary evidence that PRAME expression increases during LUAD metastatic progression. Such a stepwise increase pattern (normal < primary tumor < metastasis) underscores the specific involvement of PRAME in LUAD malignant progression and metastatic colonization. In addition, Kaplan–Meier survival analysis revealed that enhanced expression of PRAME was associated with poorer overall survival and progression‐free survival in LUAD patients (Figure 1I), highlighting its potential significance as a prognostic biomarker in this malignancy. Collectively, these findings indicate that PRAME is progressively upregulated during LUAD progression, particularly in metastatic lesions, and that elevated PRAME expression is associated with unfavorable clinical outcomes, supporting a potential role for PRAME in LUAD metastasis.

**FIGURE 1:**
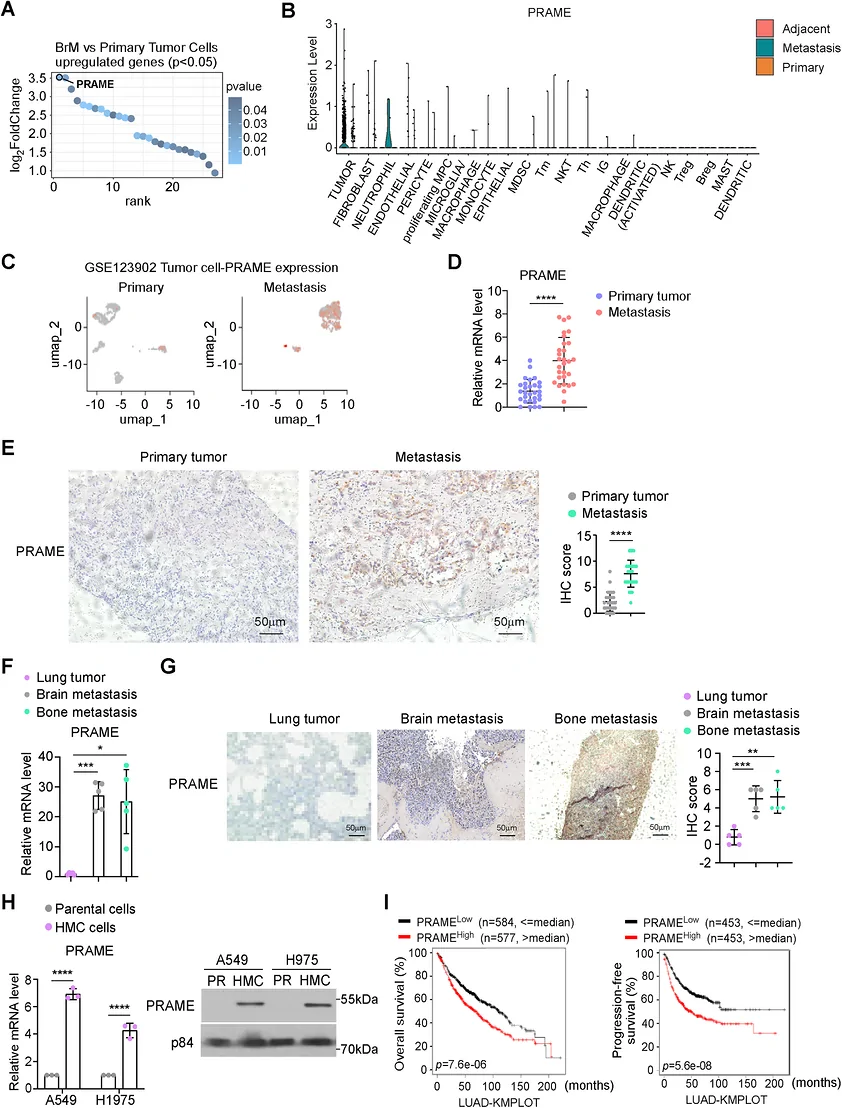
PRAME is upregulated in LUAD metastases and is associated with poor prognosis. (A) PRAME expression in metastatic versus primary tumor cells from single‐cell RNA sequencing (scRNA‐seq) dataset GSE123902. (B) PRAME expression in different cell types based on a public scRNA‐seq dataset comparing metastases with primary tumors. (C) Uniform manifold approximation and projection (UMAP) visualization of tumor cells from GSE123902, with PRAME expression levels highlighted in red. (D) qPCR validation of PRAME mRNA levels in metastases (*n = *29) and primary tumors (*n = *29). (E) Immunohistochemistry (IHC) analysis and quantification of PRAME in lung adenocarcinoma (LUAD) primary tumors (*n = *29) and metastases (*n = *29). Scale bar: 50 µm. (F and G) Validation of PRAME levels using qPCR (F) and IHC (G) in lung tumors 28 days after intracardiac injection of A549 cells, and in brain or bone metastases at the same time point in Balb/c‐nu mice (*n = *5 per group). Scale bar: 50 µm. (H) qPCR and WB validation of PRAME expression in HMC LUAD cell lines. (I) Overall survival and progression‐free survival analyses of LUAD patients using Kaplan–Meier plotter (KMplotter). Patients were divided into PRAME high‐ and low‐expressing groups based on the median PRAME mRNA level in primary tumors.

### 2.2 PRAME Endows Tumor Cells With Potent Metastatic Traits

The observed PRAME upregulation in metastatic LUAD cells prompted us to further investigate whether it could promote metastasis‐related traits in tumor cells. By using two LUAD cell lines (A549 and H1975) stably overexpressing or depleted for PRAME (Figure 2A), RNA sequencing was performed, followed by gene set enrichment analysis (GSEA). Our results showed that the metastasis‐related gene signature was enriched in cells ectopically expressing PRAME (Figure 2B), and that LUAD cells with high PRAME exhibited a more typical mesenchymal‐like morphology (Figure S2A). PRAME overexpression downregulated the epithelial marker E‐cadherin but increased the mesenchymal marker Vimentin, whereas silencing PRAME resulted in the opposite effects (Figure 2C). Moreover, PRAME expression in patients with metastasis positively correlated with EMT signature expression (Figure S2B), suggesting that PRAME‐high LUAD cells may undergo epithelial–mesenchymal transition (EMT) that is usually associated with the invasive and metastatic capacities of cancer cells. We also performed targeted correlation analyses using the TCGA–LUAD cohort, with particular focus on patients with advanced or metastatic disease, given that PRAME is an oncofetal antigen predominantly upregulated during malignant progression. Our analysis demonstrated a statistically significant positive correlation between *PRAME* mRNA expression and mesenchymal markers VIM (Vimentin) and CDH2 (N‐cadherin) in these patients (Figure S2C). These clinical data support the notion that PRAME might promote the acquisition of mesenchymal features, a hallmark of invasive LUAD cells. In contrast, although PRAME showed a negative correlation trend with CDH1 (E‐cadherin), this association did not reach statistical significance (Figure S2C). We speculate that this may be attributable to tumor heterogeneity and the complex multilayered regulation of E‐cadherin expression, including post‐transcriptional and post‐translational mechanisms, as previously reported [29, 30]. Collectively, the above data suggest an association between PRAME and EMT in advanced LUAD. Consistently, PRAME enhanced the motility of LUAD cells in vitro as demonstrated by Transwell assays (Figure 2D). To further determine whether endogenous PRAME is required for the maintenance of metastatic traits, we silenced PRAME in HMC cells, which express elevated levels of PRAME. PRAME depletion markedly suppressed metastasis‐associated malignant phenotype (Figure S2D,E), indicating that PRAME contributes to sustained aggressiveness of metastatic LUAD cells.

**FIGURE 2:**
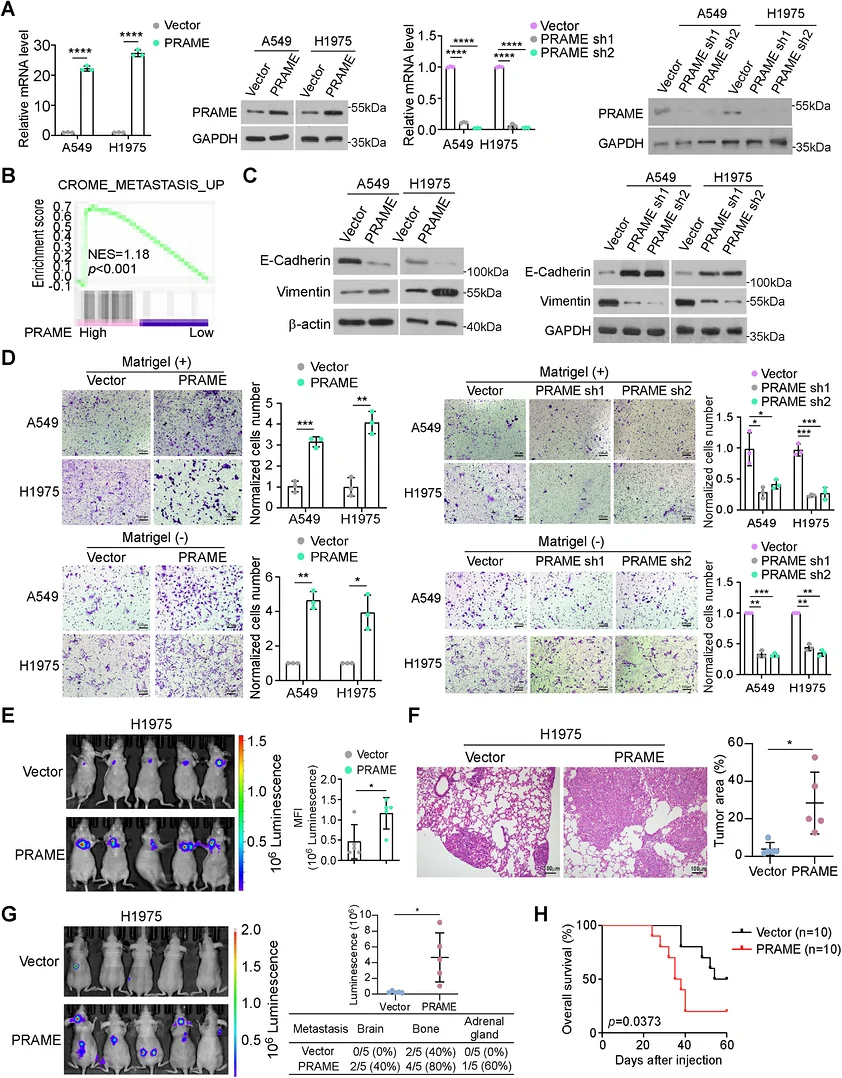
PRAME endows tumor cells with potent metastatic traits. (A) qPCR and WB validation of PRAME overexpression and knockdown in LUAD cells. (B) Gene set enrichment analysis (GSEA) showing correlation between PRAME expression and metastasis‐related signatures, based on the RNA‐seq comparing PRAME‐overexpressing A549 cells with controls. (C) WB validation of epithelial (E‐cadherin) and mesenchymal (Vimentin) markers in PRAME‐overexpressing and PRAME‐knockdown LUAD cells. (D) Transwell invasion and migration assays of PRAME‐overexpressing and PRAME‐knockdown LUAD cells. Scale bar: 100 µm. (E and F) Lung colonization assays in Balb/c‐nu mice 28 days after intravenous injection of H1975 cells with PRAME overexpression. Representative bioluminescent imaging (BLI) (E) and H&E staining (F) are shown. *n = *5 mice per group. Scale bar: 100 µm. (G and H) Systemic metastasis analysis in Balb/c‐nu mice following intracardiac injection of PRAME‐overexpressing H1975 cells. Representative BLI images (*n = *5 mice per group) (G) and animal overall survival (*n = *10 mice per group) (H) are shown. Statistical analyses were performed using a two‐tailed unpaired *t*‐test. *, *p* < 0.05; **, *p* < 0.01; ***, *p* < 0.001; and ****, *p* < 0.0001. Error bars represent the mean ± SD from three independent experiments.

Next, the pro‐metastatic role of PRAME was then validated in vivo by tail vein injection of 1×10^6^ Vector or PRAME‐overexpressing H1975‐luciferase cells into Balb/c‐nu mice. Four weeks after inoculation, mice injected with PRAME‐H1975 cells formed more tumor foci in various lobes of the lungs than the vector control cells (Figure 2E,F). In addition, intracardiac injection of PRAME‐overexpressing H1975 cells led to more robust development of metastatic lesions in multiple distant organs such as the lungs, the spine, limbs, and the brain in mice and significantly shorter survival (Figure 2G,H), demonstrating the pro‐metastatic activity of PRAME in vivo.

### 2.3 PRAME Activates TGF‐β Signaling Pathway to Foster LUAD Metastasis

To further understand how PRAME promotes LUAD metastasis, we compared RNA‐seq data from metastatic and primary LUAD tissues. While previous studies have identified the role of PRAME in inhibiting RAR signaling and activating NFY transcription [31, 32], GSEA results showed that neither RAR nor NFY signaling was enriched in LUAD metastasis (Figure S3A,B). We then assessed the activities of several metastasis‐related signaling pathways and found that PRAME robustly activated TGF‐β signaling without changing the activity of several other pathways (Figure 3A). The activation of TGF‐β signaling pathway by PRAME was further evidenced by elevated phosphorylated‐SMAD3 (p‐SMAD3) and induction of EMT drivers SNAI1 and ZEB1, together with the mesenchymal marker CDH2 (Figure 3B,C). In addition, GSEA of RNA‐seq data showed that DEGs in the TGF‐β signaling pathway were significantly enriched in PRAME‐ A549 when compared with the Vector‐A549 cells (Figure 3D). Consistently, PRAME knockdown in both parental and HMC LUAD cells hampered TGF‐β activation, reduced p‐SMAD3 levels, and decreased expression of the downstream genes (Figure 3E–J). Of particular note is that inhibition of TGF‐β signaling with SIS3, a specific inhibitor of SMAD3 [33], markedly abolished the PRAME‐enhanced invasive and migratory abilities of LUAD cells in vitro (Figure 3K). Consistently, treatment with the TGF‐β receptor inhibitor SB‐431542 or SMAD3 siRNA knockdown significantly reversed PRAME‐induced metastasis‐related traits (Figure 3L,M), further suggesting that TGF‐β/SMAD3 signaling contributes to the pro‐metastatic effect of PRAME. To evaluate whether TGF‐β inhibition can block PRAME‐driven metastasis in vivo, we performed both tail vein lung metastasis and intracardiac injection assays using H1975 cells, and administered SB‐431542 systemically to assess its effect on distal and systemic metastatic dissemination. Bioluminescence imaging revealed that while PRAME markedly increased metastatic burden in the lungs and throughout the body, TGF‐β inhibition significantly attenuated both distal and systemic metastatic signals (Figure S3C,D), demonstrating that TGF‐β pathway activation is functionally pivotal for PRAME‐mediated metastasis in vivo.

**FIGURE 3:**
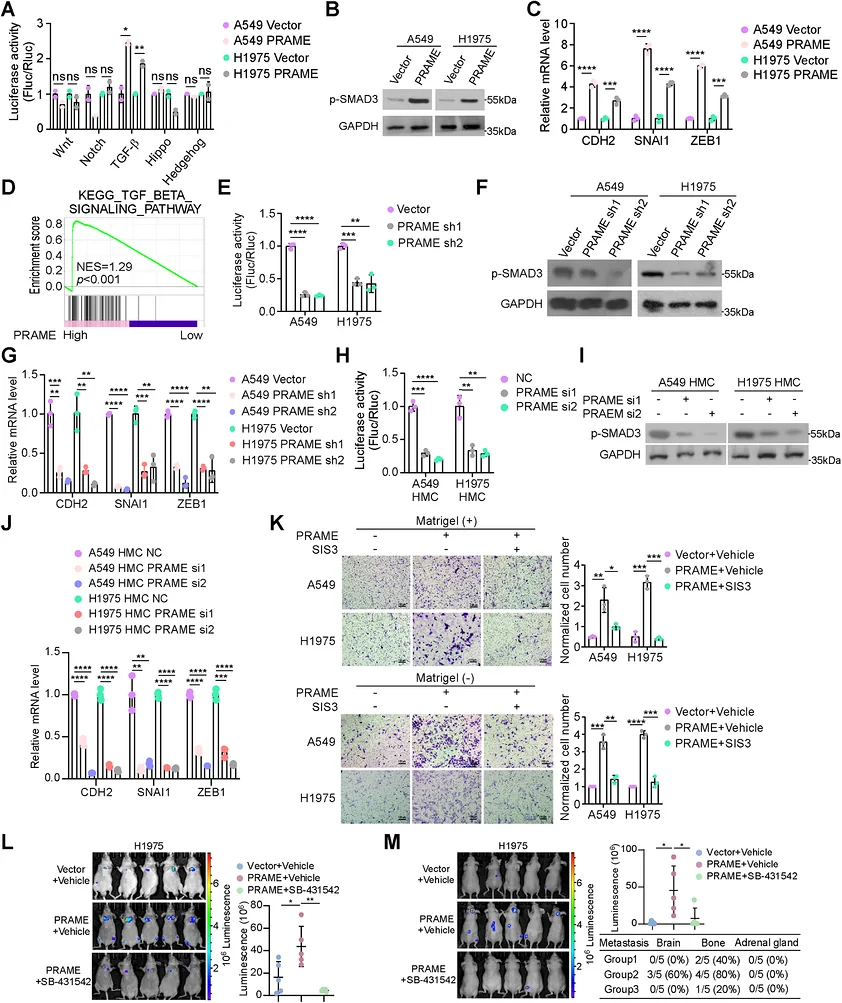
PRAME activates the TGF‐ß signaling pathway to foster LUAD metastasis. (A‐C) Validation of TGF‐β signaling activation in PRAME‐overexpressing LUAD cells. Luciferase reporter assay for various signaling pathways (A), WB validation of p‐SMAD3 levels (B), and qPCR validation of TGF‐β downstream gene expression (C). (D) GSEA showing correlation between PRAME expression and TGF‐ß signaling signature in RNA‐seq data comparing PRAME‐overexpressing A549 cells with controls. (E‐G) Validation of TGF‐β signaling in PRAME‐knockdown LUAD cells. Luciferase reporter assay (E), WB of p‐SMAD3 (F), and qPCR of downstream gene expression (G). (H‐J) Validation of TGF‐β signaling in PRAME‐knockdown HMC cells. Luciferase reporter assay (H), WB of p‐SMAD3 (I), and qPCR of downstream genes (J). (K) Invasion and migration assays of PRAME‐overexpressing LUAD cells under indicated treatment. Scale bar: 100 µm. (L) Lung colonization of H1975 cells with SB‐431542 treatment (5mg/kg) after intravenous injection in Balb/c‐nu mice. Representative BLI images on day 28 post‐inoculation are shown. *n = *5 mice per group. (M) Systemic metastasis analysis in Balb/c‐nu mice following intracardiac injection of H1975 cells with SB‐431542 treatments (5mg/kg). Representative BLI images on day 28 post‐inoculation are shown. *n = *5 mice per group. Statistical analyses were performed using a two‐tailed unpaired *t*‐test. *, *p* < 0.05; **, *p* < 0.01; ***, *p* < 0.001; and ****, *p* < 0.0001. Error bars represent the mean ± SD from three independent experiments. ns, not significant.

### 2.4 PRAME Interacts With METTL3 to Facilitate Its Ubiquitination on K235 via K63‐Linkage

As PRAME had been initially identified as a substrate recognition subunit of E3 ubiquitin ligase Cullin2 [31], and their interaction caused K48‐linked ubiquitination, leading to degradation of target proteins [34], we thus sought to examine whether PRAME's enhancing effects on TGF‐β signaling and LUAD metastasis could be associated with its impact on protein stability. To this end, mass spectrometry (MS) was performed with Flag‐PRAME immunoprecipitates of the HMC cells and exhibited that METTL3 was a potential binding partner of PRAME (Figure 4A and Table S2), which was then verified by co‐immunoprecipitation (co‐IP) analysis on endogenous proteins in cell lysates (Figure 4B). To further validate whether this interaction is direct, we performed an in vitro GST pull‐down assay using purified recombinant proteins, and our data revealed that indeed METTL3 directly binds PRAME in the absence of intermediary proteins (Figure 4C). Given that PRAME is a substrate recognition subunit of E3 ubiquitin ligase [31], we investigated its potential influence on METTL3 ubiquitination. Our results showed that PRAME drastically elevated ubiquitination of METTL3 (Figure 4D), and such an elevation was K63 linkage‐specific but independent of linkages at other lysine sites, including K6‐, K11‐, K27‐, K29‐, K33‐, and the previously reported K48 (Figures 4E and S4A). The same results were found in HMC LUAD cells, which express high levels of PRAME (Figure 4F,G). Moreover, silencing PRAME markedly reduced endogenous METTL3 ubiquitination (Figure 4H). Also of note was that overexpressing the mutant form of ubiquitin (K63R), which selectively disrupts ubiquitin chain assembly through K63 linkage, potently reduced the K63‐linked ubiquitination level of METTL3 protein in HMC cells (Figure 4I), suggesting that METTL3 can be ubiquitinated via K63 linkage. In agreement with a previous study reporting that PRAME's interaction with E3 ubiquitin ligase Cullin2 causes K48‐ instead of K63‐linked ubiquitination of substrate [34], knockdown of other Cullin2 complex components, including CUL2, ELOB, or ELOC, did not lead to comparable effects on METTL3 protein expression (Figure S4B). In addition, treatment with the broad‐spectrum deubiquitinating enzyme (DUB) inhibitor PR‐619 affected METTL3 expression (Figure 4J), suggesting that an additional upstream ubiquitination mechanism might contribute to maintaining METTL3 stability.

**FIGURE 4:**
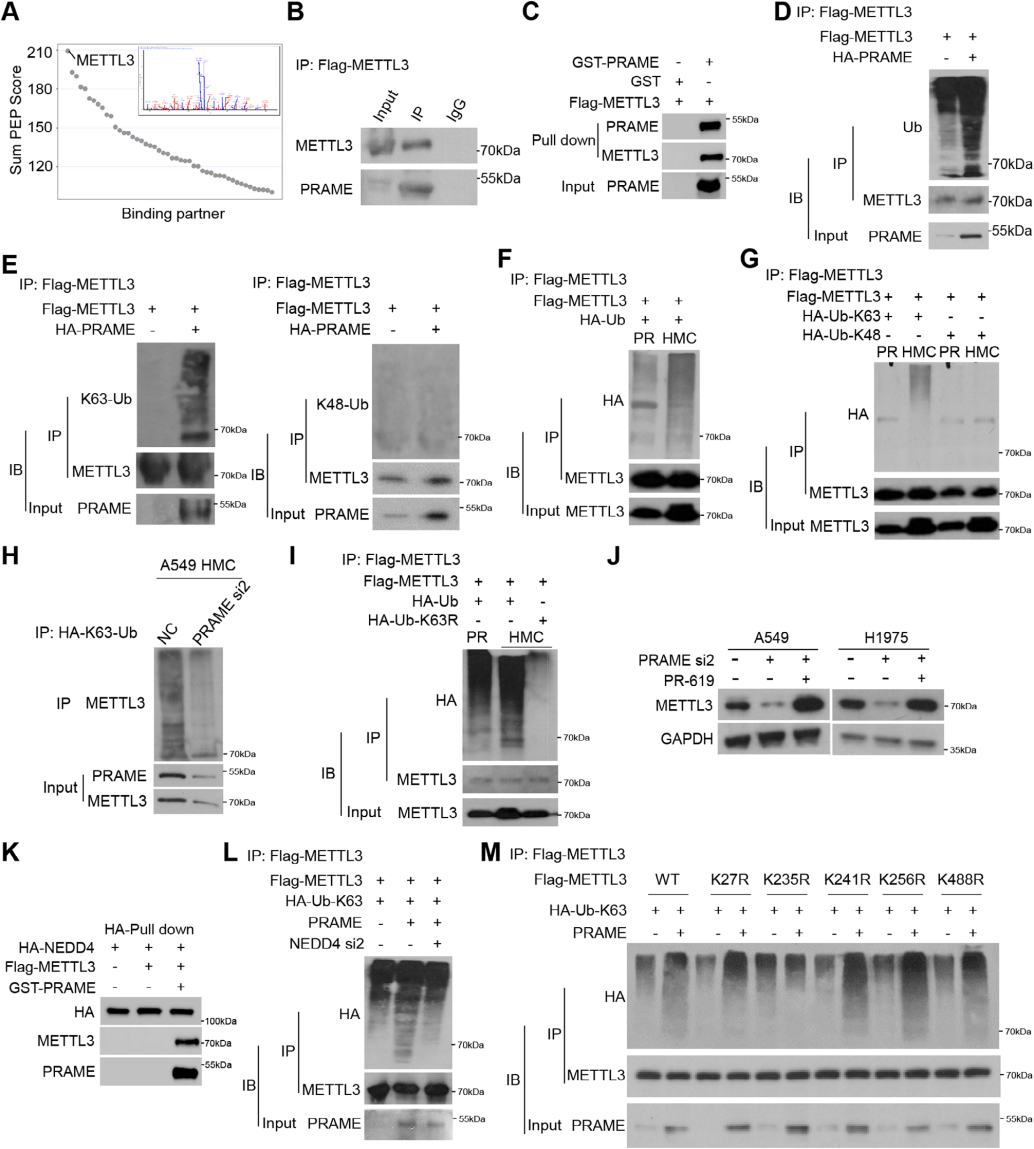
PRAME interacts with METTL3 to facilitate its ubiquitination on K235 via K63‐linkage. (A) Mass Spectrometry (MS) fingerprint of METTL3 peptide. (B) Co‐immunoprecipitation (co‐IP) analysis of the interaction between METTL3 and PRAME proteins. (C) GST‐pull‐down analysis demonstrating direct binding of recombinant Flag‐METTL3 with GST‐PRAME in vitro. (D and E) Immunoprecipitation (IP) validation of total ubiquitination (D) and K63‐/K48‐ linked ubiquitination levels (E) of indicated HEK293T cells. (F–G) IP validation of ubiquitination levels in parental (PR) and HMC A549 cells treated with MG132 (10 µm). (H) IP validation of METTL3 ubiquitination in HMC A549 cells with or without PRAME knockdown. (I) IP validation of ubiquitination in cells expressing K63R mutant ubiquitin (Ub). (J) WB validation of METTL3 levels in LUAD cells with or without PR‐619 treatment (10µM). (K) Pull‐down analysis of the direct binding among recombinant Flag‐METTL3, HA‐NEDD4, and GST‐PRAME protein in vitro. (L and M) IP validation of K63‐ linked ubiquitination in indicated HEK293T cells.

Given that PRAME induces K63‐linked non‐degradative ubiquitination of METTL3, and HECT‐family E3 ligases are recognized as major executors of K63‐linked ubiquitin chain assembly, we next sought to investigate whether NEDD4, a well‐characterized K63 E3 ligase implicated in AKT, PTEN, TGF‐β signaling, cell migration, and metastasis [35, 36, 37, 38, 39, 40, 41, 42, 43, 44], could be involved in the observed PRAME‐associated METTL3 K63 ubiquitination. Of note, our co‐IP assay found that NEDD4 indeed interacted with both PRAME and METTL3 (Figure S4C), and further analyses revealed that METTL3 does not directly bind NEDD4 under basal conditions; instead, PRAME robustly enhances the association between METTL3 and NEDD4, indicating a possibility that PRAME might function as a molecular scaffold for the NEDD4/METTL3 binding (Figure 4K). Consistently, NEDD4 depletion dramatically abolished PRAME‐elevated K63‐linked ubiquitination of METTL3 (Figure 4L). In addition, PRAME depletion remarkably impaired the K63‐linked ubiquitination of METTL3 induced by NEDD4 (Figure S4D), further supporting a pivotal role of PRAME in facilitating NEDD4‐dependent ubiquitination. These data suggest that PRAME increases METTL3 ubiquitination via K63‐linkage in NEDD4 E3 ligase‐dependent manner. To further identify the residues on METTL3 key to its K63 ubiquitination, we separately mutated five potential ubiquitinated lysine residues (K27, K235, K241, K256, and K488) on METTL3 to arginine [45] and found that lysine–arginine substitution of K235 (K235R) significantly diminished the K63‐linked ubiquitination increased by PRAME, while mutations of the other four lysine residues did not change the ubiquitinated‐METTL3 level (Figure 4M), suggesting that K235 is a key residue on METTL3 for its K63‐linked ubiquitination by PRAME. Notably, cross‐species sequence alignment revealed that K235 is highly conserved across human, mouse, and rat (Figure S4E), consistent with its functional relevance in METTL3 regulation.

### 2.5 PRAME Stabilizes METTL3 and Promotes Its Binding to WTAP or METTL14 via K63‐Linked Ubiquitination

K63‐type polyubiquitination on substrate proteins by NEDD4 typically results in their stabilization [35]. We then asked whether PRAME‐mediated ubiquitination on METTL3 via K63‐linkage is associated with METTL3 protein stability. Our results showed that ectopic expression of PRAME in parental LUAD cells significantly upregulated METTL3 protein without changing its mRNA levels (Figure 5A), and PRAME dramatically prolonged the protein half‐life of METTL3 (Figure 5B). By contrast, silencing PRAME in HMC cells exhibited the opposite effect (Figure S5A). Moreover, an increased half‐life of METTL3 protein was observed in HMC cells in which PRAME was upregulated, while disrupting ubiquitin chain assembly through K63 linkage significantly reduced METTL3 half‐life (Figure S5B,C), suggesting that such a modification might be important for METTL3 stabilization. Also of note was that the expression and half‐life of METTL3 protein increased by PRAME can be potently abrogated by NEDD4 depletion (Figure 5C,D), indicating that K63‐linked ubiquitination of METTL3 on the K235 residue is key to its protein stability promoted by PRAME. To directly assess the requirement of endogenous METTL3 K235 ubiquitination in a more physiologically relevant context, endogenous METTL3 was depleted using shRNAs targeting the 3′UTR region, followed by rescue with WT‐METTL3 or K235R‐METTL3 constructs (Figure S5D,E). Compared with WT METTL3, the K235R mutant abolished the PRAME‐induced increase in METTL3 stability (Figure 5E), suggesting that K63‐linked ubiquitination of METTL3 at K235 is essential for its stabilization by PRAME.

**FIGURE 5:**
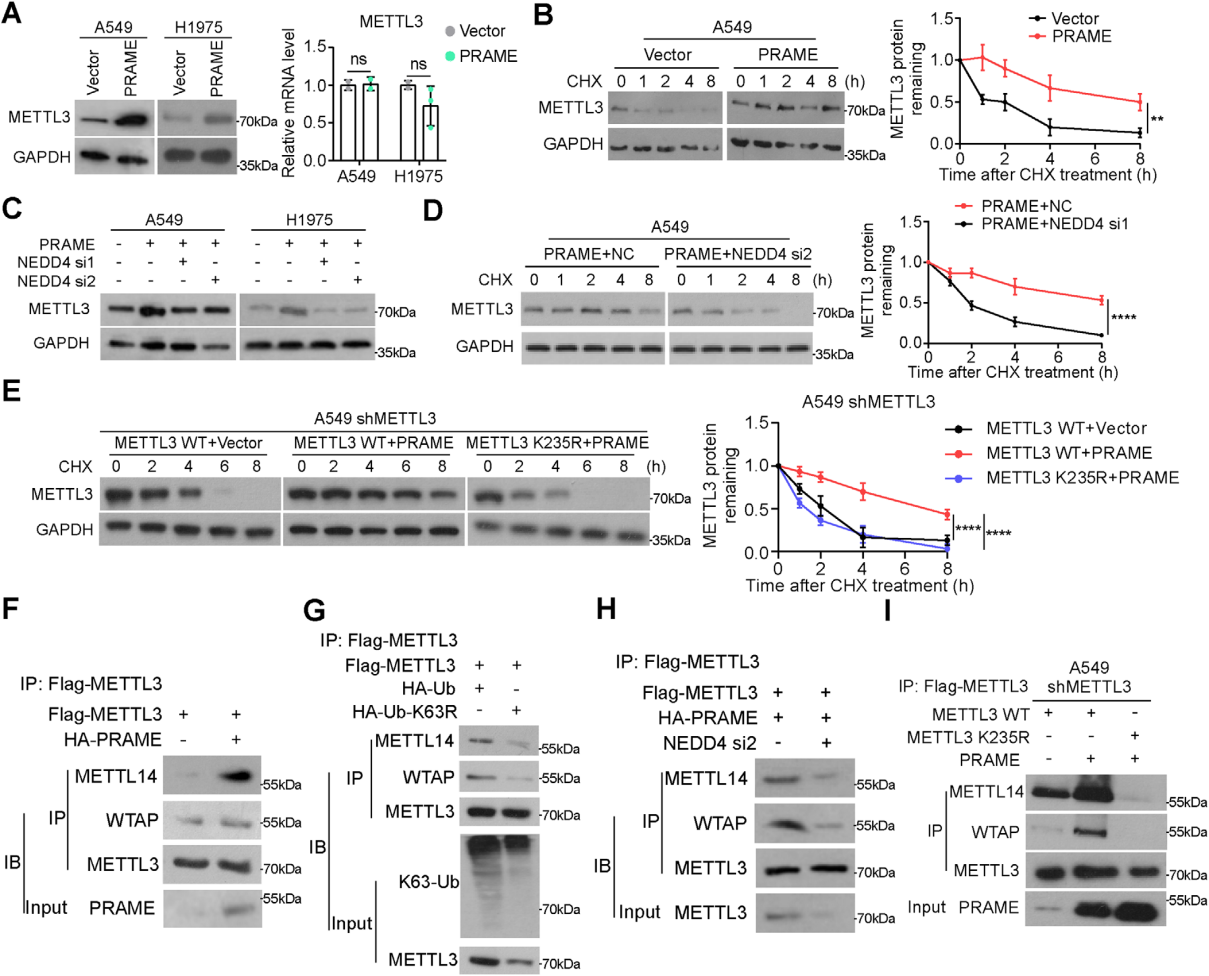
PRAME stabilizes METTL3 and promotes its interaction with WTAP or METTL14 via K63‐linked ubiquitination. (A) WB and qPCR validation of METTL3 protein levels in PRAME‐overexpressing LUAD cells. (B) METTL3 stability and quantifications in PRAME‐overexpressing A549 cells treated with cycloheximide (CHX, 10 µg/ml) for indicated time points. (C) WB validation of METTL3 levels in PRAME‐overexpressing LUAD cells with NEDD4 knockdown. (D) METTL3 stability and quantifications in PRAME‐overexpressing A549 cells with or without NEDD4 knockdown in the presence of CHX for the indicated time points. (E) METTL3 stability and quantifications in METTL3 knockdown A549 cells transfected with PRAME‐overexpressing and either wild‐type (WT) or K235R mutant METTL3 in the presence of CHX for indicated time points. (F–I) co‐IP analysis of Flag‐METTL3 interactions with methyltransferase complex (MTC) components in indicated HEK293T cells. Representative images from three experiments are shown. Statistical analyses were performed using a two‐tailed unpaired *t*‐test in (A) and two‐way ANOVA in (B), (D) and (E). **, *p* < 0.01; ***, *p*< 0.001; and ****: *p*< 0.0001. Error bars represent the mean ± SD from three independent experiments. ns, not significant.

In light of the fact that METTL3 is able to interact with METTL14 and WTAP to form the methyltransferase complex (MTC), which contributes to the m^6^A modifications on mRNAs [46, 47], we were prompted to assess whether the PRAME‐induced K63‐linked ubiquitination promotes the METTL3 interaction with the other two core components of the MTC. As expected, the PRAME upregulation enhanced the interaction between METTL3 and either METTL14 or WTAP (Figure 5F), but the K63R mutant ubiquitin markedly abolished the METTL3 binding to the above two MTC components (Figure 5G). Furthermore, the enhanced binding capacity of METTL3 with METTL14/WTAP can also be abrogated by either NEDD4 depletion or the K235R mutant (Figure 5H,I). The above findings together indicate that K63‐linked ubiquitination is functionally associated with the PRAME‐promoted METTL3 interaction with other MTC components.

### 2.6 PRAME‐Promoted TGF‐β Activation and LUAD Metastasis Require K63‐Linked Ubiquitination of METTL3

Next, we sought to elucidate whether the activation of the TGF‐β pathway and the promotion of metastasis by PRAME depend on K63‐linked ubiquitination of METTL3. Our data showed that the PRAME‐enhanced luciferase reporter activity, p‐SMAD3 level, and expression of downstream target genes were all abolished by METTL3 depletion (Figures 6A,B and S6A). Moreover, a catalytically inactive mutant METTL3 (aa395‐398, DPPW/APPA) [48] failed to facilitate PRAME‐activated TGF‐β signaling in LUAD cells (Figures 6C,D and S6B), indicating that METTL3 and its methyltransferase activity are required for the activation of TGF‐β signaling by PRAME. Of note, the enhanced luciferase reporter activity, elevation of the p‐SMAD3 level, and increased expression of the downstream target genes were all dampened by either METTL3 K235R mutation or silencing E3 ligase NEDD4 (Figures 6E,F and S6C,D), suggesting that the activation of TGF‐β signaling by PRAME depends on K63‐linked ubiquitination of the METTL3 protein.

**FIGURE 6:**
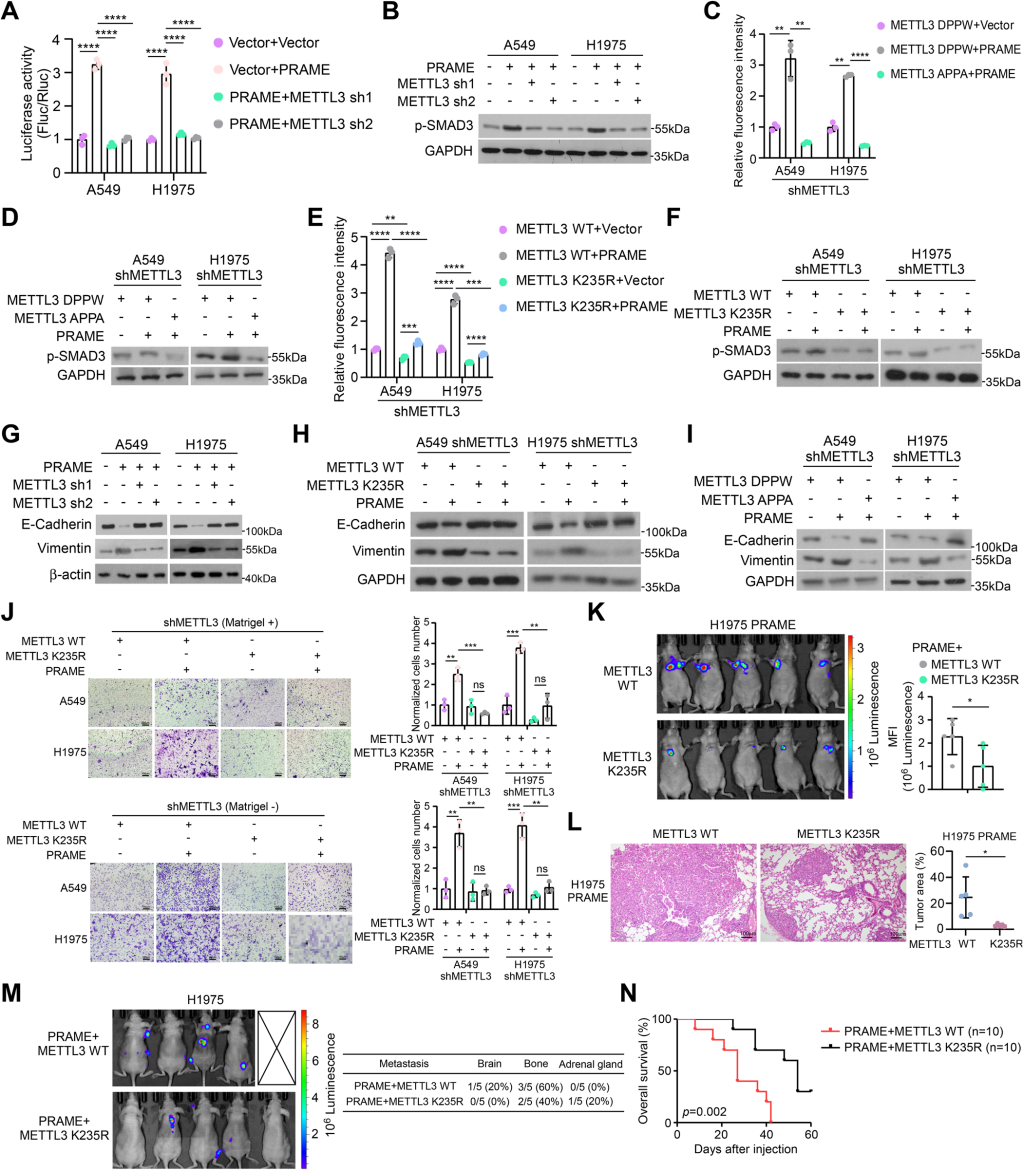
PRAME‐promoted TGF‐ß activation and LUAD metastasis require K63‐linked ubiquitination of METTL3. (A and B) Luciferase reporter assay (A) and WB (B) validation of the TGF‐ß pathway activation in PRAME‐overexpressing LUAD cells with METTL3 knockdown. (C and D) Luciferase reporter assay (C) and WB (D) validation of the TGF‐ß pathway activation in PRAME‐overexpressing LUAD cells expressing a catalytically inactive METTL3 mutant. (E and F) Luciferase reporter assay (E) and WB (F) validation of the TGF‐ß pathway activation in PRAME‐overexpressing LUAD cells expressing METTL3 K235R mutant. (G and H) WB validation of epithelial (E‐cadherin) and mesenchymal (Vimentin) markers in PRAME‐overexpressing LUAD cells with METTL3 knockdown (G) or K235R mutant (H). (I and J) WB validation of epithelial‐mesenchymal transition (EMT) markers (I) and invasion/migration assays (J) in PRAME‐overexpressing LUAD cells expressing METTL3 K235R mutant. Scale bar: 100 µm. (K and L) Intravenous injection of PRAME‐overexpressing H1975 cells expressing the METTL3 K235R mutant with METTL3 mutation into Balb/c‐nu mice and lung colonization analysis on day 28 post‐inoculation. BLI images (K) and H&E staining (L) are shown. *n = *5 mice per group. Scale bar: 100 µm. (M and N) Intracardiac injection of PRAME‐overexpressing H1975 cells expressing the METTL3 K235R mutant intoBalb/c‐nu mice and systemic metastasis analysis on day 28. BLI images (*n = *5 mice per group) (M) and overall survival (*n = *10 mice per group) (N) are shown. Statistical analyses were performed using a two‐tailed unpaired *t*‐test. *, *p* < 0.05; **, *p* < 0.01; ***, *p* < 0.001; and ****, *p* < 0.0001. Error bars represent the mean ± SD from three independent experiments.

We also investigated whether the promotion of metastasis by PRAME requires ubiquitination of METTL3 and its m^6^A catalytic activity, and found that either METTL3 depletion or METTL3 mutant lacking m^6^A catalytic activity drastically suppressed the PRAME‐promoted metastatic traits (Figures 6G,H and S6E,F). In addition, METTL3 K235R mutant remarkably abrogated the pro‐metastatic effects of PRAME, as evidenced by changes in EMT markers and decreased cell motility in vitro, as well as diminished distant metastasis and prolonged mice survival in vivo (Figure 6I–N). To assess the functional relevance of the upstream E3 ligase, we examined the role of NEDD4 in the context. Knocking down NEDD4 phenocopied PRAME loss, altering EMT markers and reducing basal invasion and migration (Figure S6G,H), while silencing NEDD4 in PRAME‐overexpressing cells largely abolished the enhancement of migration, invasion, and EMT changes driven by PRAME (Figure S6I,J). The above findings indicate that PRAME might facilitate LUAD metastasis‐related traits through mediating K63‐linked ubiquitination of METTL3 and requires its m^6^A methyltransferase activity.

### 2.7 The Effects of PRAME Depend on m^6^A Modification on *TGFB2* mRNA by METTL3

To elucidate how TGF‐β signaling is activated and metastasis is augmented by PRAME via METTL3 ubiquitination, we examined the expressions of several important regulators for this pathway, such as *TGFB1*, *TGFB2*, *TGFB3*, *TGFBR1*, *TGFBR2*, *SMAD2*, *SMAD3*, and *SMAD4*, and found that the *TGFB2* ligand was the most prominently upregulated one in response to PRAME overexpression (Figure 7A), and TGF‐β2 protein was indeed elevated in the supernatant of cultured PRAME‐overexpressed LUAD cells (Figure 7B). Furthermore, analyses of the public m^6^A‐seq data of H1299 LUAD cells revealed a specific m^6^A modification peak in the 5’UTR of *TGFB2* mRNA (Figure 7C), and this observation was confirmed by RIP‐qPCR assay in the A549 LUAD cell line (Figure 7D). Importantly, MeRIP–qPCR assay demonstrated that PRAME overexpression significantly increased m^6^A modification on *TGFB2* 5’UTR (Figure 7E). We next interrogated the mechanistic link between PRAME and TGFB2 regulation. PRAME directly interacts with METTL3 and promotes K63‐linked ubiquitination at K235, enhancing METTL3 stability and assembly into the methyltransferase complex with METTL14/WTAP. Enhanced METTL3 activity increases m^6^A modification on *TGFB2* mRNA, which is recognized by the m^6^A reader IGF2BP3, which had been previously found to play an important role in LUAD metastasis via stabilizing target mRNA [26]. Consistently, depletion of METTL3, expression of catalytic‐dead METTL3 (DPPW/APPA), or the K235R mutant, as well as silencing IGF2BP3, attenuated PRAME‐induced *TGFB2* upregulation (Figures 7F–I and S7A–D). Furthermore, overexpression of PRAME prolonged the half‐life of *TGFB2* mRNA, whereas METTL3 K235R mutation reversed this effect (Figure 7J,K), indicating that K63‐linked ubiquitination of METTL3 is required for *TGFB2* transcript stabilization. The above findings suggest that PRAME regulates *TGFB2* expression in an m^6^A‐dependent manner, requiring K63‐linked ubiquitination of METTL3. TGFB2 was essential for PRAME‐driven TGF‐β activation and metastatic traits. *TGFB2* knockdown reversed PRAME‐induced activation of TGF‐β/SMAD3 signaling and abrogated PRAME‐induced LUAD cell invasion and migration (Figure 7L,M), further suggesting that TGFB2 is essential for PRAME‐facilitated TGF‐β activation and LUAD metastasis. Moreover, knockdown of IGF2BP3 reversed PRAME‐mediated activation of TGF‐β signaling, upregulation of p‐SMAD3, downstream target gene expression, EMT phenotypic changes, and metastatic behaviors (Figure S7E–H), indicating that IGF2BP3 is essential for mediating PRAME‐driven TGF‐β pathway activation and pro‐metastatic traits. Taken together, these results demonstrate that PRAME regulates *TGFB2* expression and TGF‐β pathway activation in an m^6^A‐dependent manner, requiring K63‐linked ubiquitination of METTL3 and the m^6^A reader IGF2BP3.

**FIGURE 7:**
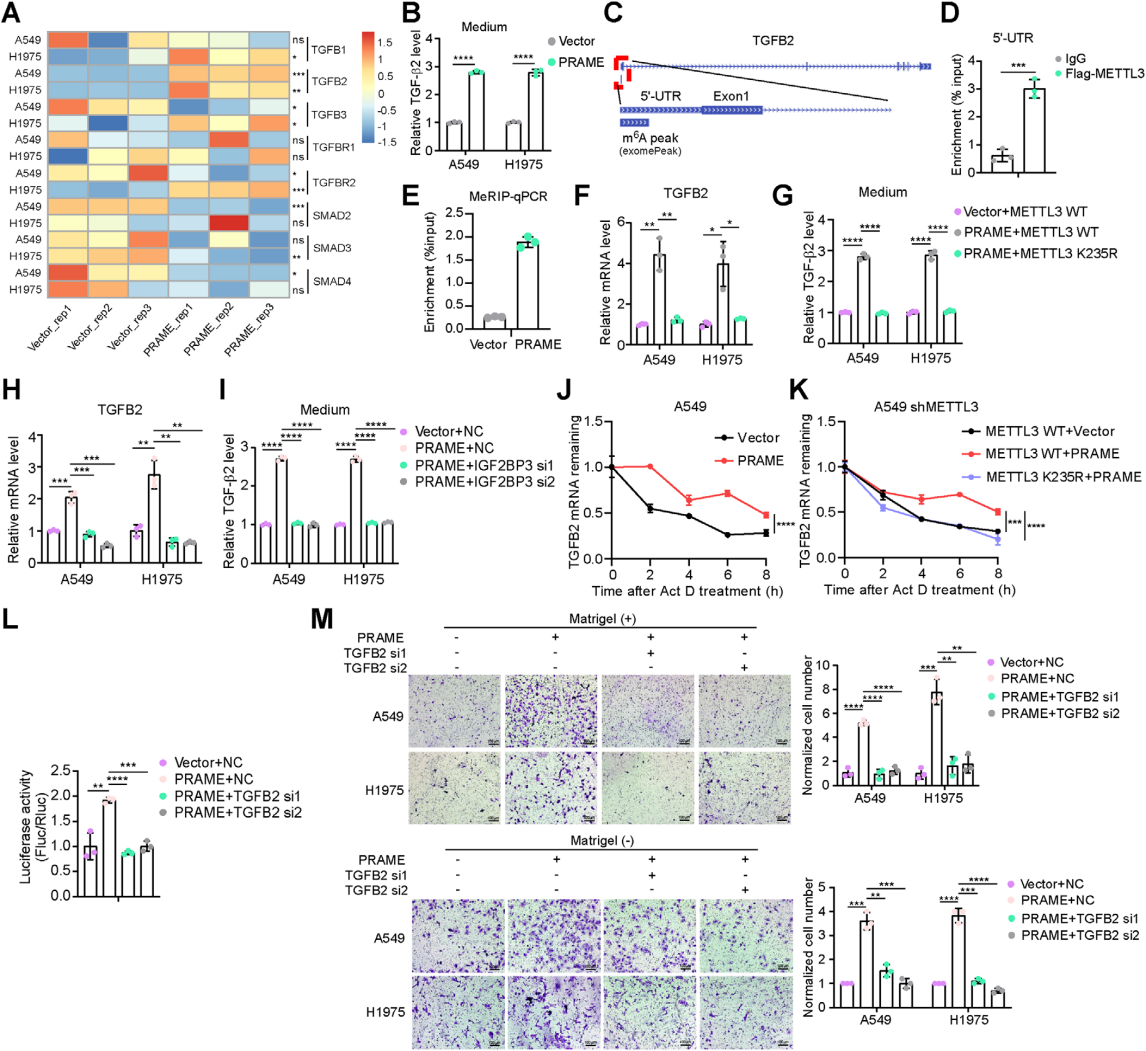
The effects of PRAME depend on m6A modification of TGFB2 mRNA by METTL3. (A) qPCR validation of TGF‐ß pathway‐related gene mRNA levels in PRAME‐overexpressing LUAD cells. (B) Validation of TGFB2 secreted protein levels in the cellular supernatant from PRAME‐overexpressing LUAD cells by enzyme‐linked immunosorbent assay (ELISA). (C) m^6^A modification profile of *TGFB2* mRNA in LUAD cells based on the public web tool REPIC (RNA Epitranscriptome Collection). Peak calling was performed by exomePeak. (D) RNA immunoprecipitation quantitative polymerase chain reaction (RIP‐qPCR) detecting the interaction between METTL3 protein and the 5’‐UTR of *TGFB2* mRNA in A549 cells. (E) Methylated RNA immunoprecipitation quantitative polymerase chain reaction (MeRIP–qPCR) assay showing enrichment of m^6^A modification in the *TGFB2* 5’UTR in A549 cells overexpressing vector control or PRAME. (F and G) qPCR (F) and ELISA (G) validation of *TGFB2* mRNA and secreted protein levels in PRAME‐overexpressing LUAD cells with METTL3 K235R mutant. (H and I) qPCR (H) and ELISA (I) validation of *TGFB2* mRNA and secreted protein levels in PRAME‐overexpressing LUAD cells with IGF2BP3 knockdown. (J and K) qRT‐PCR assays showing *TGFB2* mRNA stability in the indicated A549 cells. (L) Luciferase reporter assay validation of TGF‐ß signaling activity in PRAME‐overexpressing LUAD cells with *TGFB2* knockdown. (M) Invasion and migration assays of PRAME‐overexpressing LUAD cells with *TGFB2* knockdown. Scale bar: 100 µm. Representative images from three experiments are shown. Statistical analyses were performed using a two‐tailed unpaired *t*‐test. *, *p* < 0.05; **, *p* < 0.01; ***, *p* < 0.001; and ****, *p* < 0.0001. Error bars represent the mean ± SD from three independent experiments.

### 2.8 PRAME Upregulation is Associated With Promoter Hypomethylation and Links to Activation of the METTL3/TGF‐β Signaling Axis

To further understand the effect of PRAME upregulation on METTL3 protein stability and TGF‐β activation in metastatic LUAD, we examined their expression in patient samples. Results showed that compared to that in primary tumors, METTL3 protein expression was significantly higher in metastatic lesions where PRAME levels were also elevated (Figure 8A). Moreover, increased levels of METTL3 protein were also observed in metastatic tumors developed by intracardiac injection of the A549 LUAD cells in BALB/c‐nu mice (Figure 8B). Furthermore, in two LUAD HMC cell lines with HMC, a dramatic increase of METTL3 protein expression in multiple distant sites was detected (Figure 8C), further supporting the notion that METTL3 protein is upregulated in LUAD metastases. Nevertheless, *METTL3* mRNA expression in LUAD metastases, tumor cells from distant metastatic sites, and the HMC cell lines was not significantly elevated when compared with that in their respective control counterparts, that is, in primary tumors, in tumor cells isolated from primary LUAD, and in the parental cell lines (Figure S8A–D), demonstrating increased protein rather than mRNA levels of *METTL3* in metastatic lesions. Notably, through IHC analysis, we also found significant increases of both TGF‐β2 and p‐SMAD3 in clinical metastatic lesion samples compared with primary tumors (Figure 8D), with p‐SMAD3 showing strong nuclear localization specifically in PRAME‐high lesions, indicating activation of the TGF‐β signaling pathway in metastatic LUAD. Like PRAME, increased expression of *TGFB2* was associated with poorer overall survival and progression‐free survival in LUAD patients (Figure 8E). More importantly, tumor tissues exhibiting high PRAME expression also correlatively displayed upregulated levels of METTL3, TGF‐β2, and p‐SMAD3, and vice versa (Figure 8F), indicating a tight correlation between PRAME and the activity of a signaling axis involving METTL3 and TGF‐β in clinical LUAD.

**FIGURE 8:**
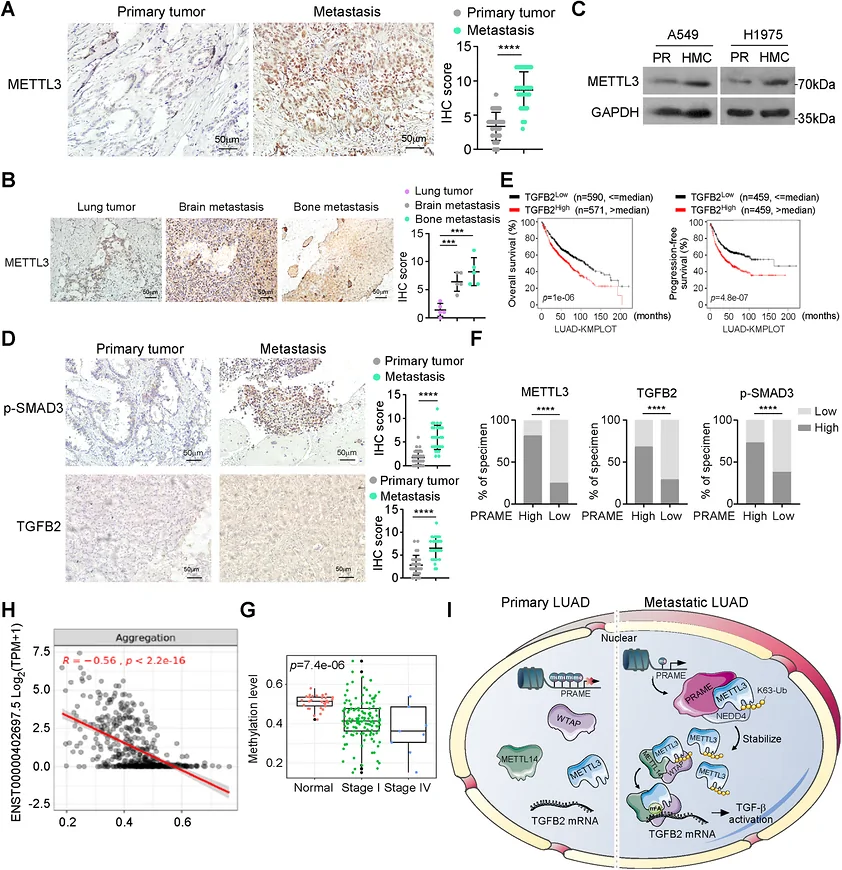
PRAME upregulation is associated with promoter hypomethylation and links to activation of the METTL3/TGF‐ß signaling axis. (A) IHC analysis and quantification of METTL3 in LUAD primary tumors (*n = *29) and metastases (*n = *29). Representative images from five random microscopic fields are shown. Scale bar: 50 µm. (B) IHC validation of METTL3 levels in lung tumors 28 days after intracardiac injection of A549 cells, and in brain or bone metastases in Balb/c‐nu mice (*n = *5 per group). Scale bar: 50 µm. (C) WB validation of METTL3 expression in HMC LUAD cell lines. (D) IHC analysis and quantification of p‐SMAD3 and TGFB2 in LUAD primary tumors (*n = *29) and metastases (*n = *29). Scale bar: 50 µm. (E) Overall survival and progression‐free survival analyses of LUAD patients based on KMplotter. Samples were classified into TGFB2 high‐ or low‐expressing groups using the median mRNA level in primary tumors as the cutoff. (F) Association between PRAME, METTL3, TGFB2, and p‐SMAD3 expression in LUAD metastases from 29 patients. Statistical analyses were performed using the Chi‐square test. (G) Analysis of PRAME DNA methylation levels in normal lung tissue and stage I or Stage IV LUAD based on the TCGA dataset. Statistical analyses were performed using ANOVA. (H) Correlation between PRAME DNA methylation levels and mRNA expression in TCGA–LUAD dataset. (I) Schematic diagram illustrating the proposed PRAME/METTL3/TGF‐ß signaling axis in LUAD metastasis. Statistical analyses were performed using a two‐tailed unpaired *t*‐test. ***, *p* < 0.001; and ****, *p* < 0.0001. Error bars represent means ± SD from three independent experiments. ns, not significant.

As hypomethylation has been previously reported as a mechanism underlying PRAME upregulation [49], we next attempted to determine whether PRAME upregulation in metastases of LUAD patients is relevant to the methylation status of the PRAME gene. Using the TCGA dataset, we found that tumor samples derived from Stage IV LUAD patients (advanced stage with distant metastasis) exhibited lower DNA methylation levels on the PRAME promoter when compared with normal human samples or those from Stage I LUAD (Figure 8G), and the methylation levels of these sites were conversely correlated with PRAME expression (Figure 8H), indicating that DNA hypomethylation might contribute to the observed increased expression of PRAME in LUAD metastasis.

Taken together, the current study found that PRAME, upregulated possibly due to promoter hypomethylation in metastatic LUAD, facilitates K63‐linked ubiquitination of METTL3, thereby enhancing its protein stability and interaction with METTL14 and WTAP, resulting in increased m^6^A modification on *TGFB2* mRNA, activation of TGF‐β signaling, and promotion of LUAD metastasis (Figure 8I).

## 3 Discussion

While indicated in the pathogenesis of LUAD metastasis [15, 16, 17], the precise biological role of PRAME in the disease remains unclear, and the molecular mechanism underlying the pro‐metastatic effects of PRAME is yet to be identified. In the present study, we provide robust evidence for the pro‐metastatic function of PRAME: a remarkable elevation of PRAME functionally confers aggressive metastatic properties on LUAD cells, likely via a previously unidentified mechanism by which PRAME facilitates the K63‐linked ubiquitination of METTL3 to stabilize METTL3 and enhance its interaction with METTL14 or WTAP, thus leading to m^6^A‐dependent activation of the pro‐metastatic TGF‐β signaling. Interestingly, while prior reports demonstrated that PRAME antagonizes retinoic acid (RA)‐induced receptor activation in melanoma [32] and binds transcriptionally active NFY promoters in leukemia [31], we have not observed a clear association between RAR or NFY signaling and LUAD metastasis. Notably, PRAME has been found to promote degradative ubiquitination via Cullin‐RING E3 ligases (CRLs) in the context of tumor proliferation control or general transcriptional regulation, thereby facilitating cell‐cycle progression and tumor growth [13, 50]. In contrast, our present study in the metastatic setting reveals a non‐degradative ubiquitination mechanism, which is mediated by the HECT‐type E3 ligase NEDD4. Whereas CRLs are primarily associated with K48‐linked proteolytic ubiquitination [51], HECT‐type E3 ligases like NEDD4 preferentially mediate K63‐linked non‐proteolytic ubiquitination of their substrates [52, 53]. In metastatic LUAD, PRAME associates with NEDD4, inducing K63‐linked non‐degradative ubiquitination of METTL3, which enhances the stability and functional assembly of the m^6^A writer complex. Thus, our findings highlight a novel non‐proteolytic ubiquitination function of PRAME and establish it as a critical molecular basis for LUAD metastasis. They provide new insights into the context‐dependent functions of PRAME and identify potential therapeutic targets for intervention.

There has been a long‐standing inconsistency in the reported correlation between *METTL3* expression and clinical prognosis in LUAD, with different studies using either mRNA or protein abundance as the indicator of METTL3 levels [25, 54, 55]. Our current study in metastatic LUAD, which reveals a discrepancy between METTL3 protein and mRNA levels, may at least partly explain the aforementioned inconsistency. In such a context, we identify K63‐linked ubiquitination as a novel post‐translational modification of METTL3, and demonstrate that such modification is responsible for the stabilization of METTL3 protein. Unlike other METTL3 PTMs such as SUMOylation, phosphorylation, and lactylation that may alter the METTL3's methyltransferase enzymatic activity, abilities to target translation, and to capture RNA, respectively [56, 57, 58]. Ubiquitination is uniquely directly correlated with METTL3 protein abundance. Importantly, the identified ubiquitination site K235 is highly conserved across multiple species, including human, mouse, and rat. Classically, K63‐linked ubiquitination primarily regulates signal transduction, protein complex assembly, subcellular localization, and protein–protein interactions, in contrast to K48‐linked ubiquitination, which directly mediates proteasomal degradation [45, 59, 60, 61, 62]. Based on our results, we speculate that PRAME‐induced K63 ubiquitination stabilizes METTL3 through two non‐mutually exclusive mechanisms. First, competitive occupancy of non‐degradative ubiquitin chains may attenuate the effects of degradative modifications. Although we initially tested whether K63 ubiquitination stabilizes METTL3 by inhibiting K48‐linked ubiquitination, PRAME did not significantly reduce K48 ubiquitination of METTL3. Notably, we observed that high PRAME expression markedly decreased K27‐linked ubiquitination of METTL3 while simultaneously increasing K63‐linked ubiquitination. Previous studies have revealed that K27‐linked ubiquitination can contribute to protein degradation in certain contexts [63]. We therefore propose that PRAME‐induced K63 chains may competitively occupy critical lysine residues (e.g., near K235), preventing the formation of degradative K27‐linked chains and thereby indirectly prolonging METTL3 half‐life. Secondly, K63 ubiquitination may promote assembly of protein complexes, resulting in a conformational protection effect. Our data show that K63 ubiquitination enhances METTL3 binding to METTL14 and WTAP, promoting stable assembly of the MTC. Proteins incorporated into a stable complex are often less susceptible to degradation due to shielding of critical domains, reduced exposure of degradation recognition sites, or overall conformational stabilization. This “complex‐protection effect” has been reported in multiple protein homeostasis systems [64, 65]. Thus, we propose that K63 ubiquitination stabilizes METTL3 by enhancing MTC integration, reducing its recognition by degradation machinery. In summary, PRAME‐induced K63‐linked ubiquitination, instead of acting as a direct degradative signal, probably stabilizes METTL3 through a combination of inhibiting competitive degradative ubiquitin modifications and promoting MTC assembly‐mediated structural protection, which explains the observed correlation between K63 ubiquitination and METTL3 protein accumulation in our study.

In addition to its role in stabilizing METTL3, our findings suggest that K63‐linked ubiquitination serves as a critical regulatory mechanism for the assembly of the m^6^A methyltransferase complex. We observed that disruption of K63‐linked ubiquitination—either through ubiquitin mutation (K63R), NEDD4 depletion, or mutation of the ubiquitination site on METTL3 (K235R)—markedly impaired the interaction between METTL3 and its core binding partners METTL14 and WTAP. These results indicate that K63‐linked ubiquitination is not merely a post‐translational modification affecting protein stability, but also a key determinant of METTL3 complex formation. Mechanistically, several non‐mutually exclusive models may explain how K63‐linked ubiquitination facilitates METTL3 complex assembly. For example, K63‐linked ubiquitin chains may induce conformational changes in METTL3, thereby exposing or stabilizing interaction interfaces required for binding to METTL14 and WTAP. Moreover, the non‐proteolytic nature of K63‐linked ubiquitination may allow it to function as a signaling scaffold, providing a docking platform for protein‐protein interactions, as has been reported in other signaling pathways [66, 67]. In this context, ubiquitin chains conjugated to METTL3 may directly or indirectly enhance recruitment of its cofactors. Furthermore, by prolonging the half‐life of METTL3, K63‐linked ubiquitination increases the effective pool of METTL3 available for incorporation into the methyltransferase complex, thereby promoting complex assembly in a quantitative manner. Importantly, these mechanisms are not mutually exclusive and may act in concert to ensure efficient formation of the METTL3/METTL14/WTAP complex. While our study primarily provides functional and biochemical evidence supporting this model, future structural investigation and molecular dynamics simulations will be valuable in further elucidating the precise conformational changes induced by K63‐linked ubiquitination.

In addition, our study provides a coherent framework explaining how PRAME activates the TGF‐β signaling cascade in LUAD metastasis. We demonstrate that PRAME directly interacts with METTL3 and promotes its K63‐linked ubiquitination at K235, thereby enhancing METTL3 protein stability and facilitating assembly of the METTL3/METTL14/WTAP methyltransferase complex. Importantly, our data further suggest that PRAME primarily functions as a molecular scaffold, recruiting the E3 ligase NEDD4 to METTL3 and thereby promoting NEDD4‐dependent K63‐linked ubiquitination. Enhanced METTL3 expression and binding with MTCs subsequently increase m^6^A modification on *TGFB2* mRNA, particularly within the 5’ UTR region. These m^6^A marks are recognized by the m6A reader IGF2BP3, leading to stabilization of *TGFB2* transcripts and increased secretion of TGF‐β2 ligand. Consequently, activation of downstream TGF‐β/SMAD3 signaling drives EMT and metastatic phenotypes in LUAD cells. Together, these findings establish a mechanistic PRAME–METTL3–TGFB2 axis explaining ubiquitination‐dependent regulation of the m^6^A machinery to activation of pro‐metastatic TGF‐β signaling. Nonetheless, while our data identify TGFB2/TGF‐β signaling as a dominant functional output of the PRAME/METTL3 axis, we do not exclude the possibility that additional METTL3‐dependent m^6^A targets also contribute to metastatic progression, as METTL3 broadly regulates RNA stability and across multiple oncogenic pathways [68, 69, 70, 71, 72, 73]. Why the PRAME–METTL3 regulatory axis preferentially converges on TGF‐β signaling in metastatic LUAD therefore remains an important question for future investigation. Moreover, although MeRIP‐seq consistently identified prominent m^6^A enrichment peaks within the 5′UTR region of *TGFB2* mRNA, precise validation of individual m^6^A sites by site‐specific mutagenesis remains technically challenging. The detected peak spans multiple potential DRACH consensus motifs [74], raising the possibility of functional redundancy among nearby methylation sites. Mutating a single motif may therefore be insufficient to abolish m^6^A modification, whereas simultaneous mutation of multiple motifs could disrupt local RNA secondary structure and introduce confounding effects independent of m^6^A regulation. Nevertheless, multiple complementary lines of evidence support the functional importance of *TGFB2* m^6^A modification in this study, including enrichment of m^6^A signals on *TGFB2* transcripts, the requirement of both METTL3 and IGF2BP3 for *TGFB2* expression and stability, and the essential role of TGFB2 in mediating downstream TGF‐β activation and metastatic phenotypes. Future studies using high‐resolution m^6^A mapping and precise epitranscriptomic editing approaches will help further define the contribution of individual m6A sites within *TGFB2* mRNA.

PRAME is typically absent in normal tissues but can be upregulated in cancer cells [12]. In melanoma and leukemia, expression of PRAME has been reported to be regulated via DNA hypomethylation and/or transcriptional modulation mediated by transcription factor MZF1, p53, or TEAD1 [49]. Interestingly, we in this study did not observe expressional differences of MZF1 and p53 between metastatic and primary LUAD, but found a slight increase in TEAD1 (data not shown) and hypomethylation of the PRAME promoter. Of note, aberrant promoter hypermethylation is a common event in LUAD and has been found to result in epigenetic inactivation or silencing of tumor suppressor genes such as CDKN2A, BRCAs, EPHB6, and S100A2. Demethylating therapy is therefore being tested for its potential therapeutic efficacy against lung cancer, and clinical trials investigating the methylation inhibitor 5‐aza‐2'‐deoxycitidine (Decitabine, a U.S. FDA‐approved agent) for the malignancy have been conducted (NCT02664181, NCT02608268, NCT03233724, http://www.clinicaltrials.org). In light of the fact that inhibiting methylation by 5‐aza‐2'‐deoxycitidine (decitabine) results in enhanced PRAME expression [49], the therapeutic benefit of demethylation‐induced tumor growth suppression might be counteracted by PRAME‐promoted metastasis. In fact, it has been reported that decitabine exposure could result in aggressiveness of tumors. In a phase II clinical trial on non‐small cell lung cancer (NCT02664181), treatment with Nivolumab and Decitabine does not provide therapeutic advantage compared to nivolumab use alone, in terms of overall survival and time to progression. Thus, as an alternative approach, combining demethylation with simultaneous targeting of PRAME warrants further investigation for its potential therapeutic benefit against metastatic LUAD.

Despite the above new findings, the present study has several limitations, including the need for validation in larger independent patient cohorts, more physiologically relevant metastatic models, further mechanistic validation based on structural and biochemical studies, and higher‐resolution approaches to interrogate site‐specific ubiquitination and m^6^A regulation. In addition, the strategy of combining demethylating agents with PRAME targeting to treat metastatic LUAD, as well as the feasibility, specificity, and safety of targeting PRAME in vivo, remain to be evaluated in further experimental and clinical studies. Resolving these limitations will not only strengthen the clinical and mechanistic significance of the PRAME/METTL3/TGFB2 axis, but will also facilitate the rational design of therapeutic strategies aimed at disrupting this pro‐metastatic pathway.

## 4 Conclusion

In conclusion, our study establishes a previously unrecognized PRAME–METTL3–TGFB2 regulatory axis that drives LUAD metastasis. We demonstrate that PRAME functions as a critical upstream regulator of METTL3 by promoting K63‐linked ubiquitination at K235, thereby stabilizing METTL3 and facilitating its incorporation into the m^6^A methyltransferase complex; this in turn enhances m^6^A modification and stabilization of *TGFB2* mRNA through the IGF2BP3 reader, leading to activation of TGF‐β signaling and acquisition of metastatic phenotypes. Moreover, our study is the first to show that PRAME promotes K63‐linked non‐proteolytic ubiquitination and identifies it as a previously uncharacterized post‐translational modification of METTL3. These findings reveal an unexpected link between ubiquitin signaling and epitranscriptomic regulation in LUAD metastasis and identify the PRAME/METTL3/TGFB2 axis as a potential therapeutic vulnerability in metastatic disease.

## 5 Materials and Methods

### 5.1 Tumor Specimens From Patients

Both primary and metastatic tumor specimens were retrospectively collected from patients who underwent surgical resection or biopsy at Jiangmen Central Hospital (Jiangmen, Guangdong, China) between 2013 and 2023. All patients provided written informed consent, and the study was approved by the Institutional Ethics Committee (Approval No. [2024] 226A). Histopathological features and clinical staging of LUAD specimens were assessed according to the most recent Tumor–Node–Metastasis (TNM) staging criteria established by the Union for International Cancer Control (UICC).

### 5.2 Cell Culture

The LUAD cell lines A‐549 (RRID: CVCL_0023) and NCI‐H1975 (RRID: CVCL_1511), together with the human embryonic kidney cell line HEK293T (RRID: CVCL_0063), were obtained from the cell bank of the Shanghai Institutes of Biological Sciences (Shanghai, China) or Fu Erbo Biotechnology Co., Ltd. (Guangzhou, China). Cells were maintained in Dulbecco's modified Eagle's medium (DMEM; Invitrogen, Carlsbad, CA, USA) supplemented with 10% fetal bovine serum (FBS; HyClone, Logan, UT, USA). Cell line authentication was performed by short tandem repeat (STR) profiling at the Laboratory of Forensic Medicine, Sun Yat‐sen University (Guangzhou, China). All cell cultures were routinely screened for mycoplasma contamination and confirmed to be mycoplasma‐free.

### 5.3 RNA Extraction and Real‐Time PCR (qPCR) Assay

Total RNA was extracted from cells with TRIzol reagent (Invitrogen, Carlsbad, CA, USA) according to the manufacturer's protocol. For reverse transcription, RNA templates were converted into cDNA using random primers and the GoScript Reverse Transcription Mix (Promega, Madison, WI, USA). Quantitative real‐time PCR was subsequently carried out on a CFX96 Real‐Time PCR Detection System (Bio‐Rad, Richmond, CA, USA) using 2× SYBR Green Master Mix (Promega, Madison, WI, USA). The expression levels of target genes were normalized against β‐actin, and relative changes in gene expression were calculated using the 2^−ΔΔC^_t_ method.

### 5.4 Plasmids, siRNAs, and Transfection

The coding sequences of *PRAME*, *METTL3*, and *NEDD4* were generated by PCR and subcloned into the lentiviral transfer plasmid pSin‐puro (Addgene, Cambridge, Massachusetts, USA). To deplete *PRAME* and *METTL3* expression, specific shRNA sequences (*PRAME* shRNA1: GCTGGACTCTATTGAAGATTT; shRNA2: GCTGGACTCTATTGAAGATTT; *METTL3* shRNA1: CAAGTATGTTCACTATGAA; shRNA2: ATGTTGACTTGAGGAGTTAAT; *METTL3* 3’UTR shRNA1: CGTCACTATCTTGGGCAAGTT; and *METTL3* 3’UTR shRNA2: GCAAGTATGTTCACTATGAAA) were cloned into the pSuper‐retro vector plasmid (Clontech, Palo Alto, CA, USA) to generate pSuper‐*PRAME*‐sh1/sh2 and pSuper‐*METTL3*‐sh1/sh2, respectively. siRNAs targeting *NEDD4* (si1: GGAAGGACCTATTATGTAA; si2: GGAACAACCTACACTTCCT), *TGFB2* (si1: GTCCCAAGATTTAGAACCT; si2: GCAGCACACTCGATATGGA), *CUL2* (si1: GCAAGCUACAUCGGAUGUAUATT; si2: GCAGACUAUAUGGACUGCUUATT), *ELOB* (si1: CCAACUCUUGGAUGAUGGCAATT; si2: AGAGCUGCCCGAUGUGAUGAATT), and *ELOC* (si1: CGUACAAGGUUCGCUACACUATT; si2: GCCAUGUAUGUCAAAUUGAUATT) were purchased from Ribobio (Guangzhou, China). Transfection was performed using the Lipofectamine 3000 reagent (Invitrogen, Carlsbad, CA, USA) following the manufacturer's instructions.

### 5.5 Tumor Xenografts In Vivo

All procedures involving animals were conducted in accordance with institutional guidelines and received approval from the Institutional Animal Care and Use Committee of Sun Yat‐sen University (approval number: SYSU‐IACUC‐2022‐B1723). Female BALB/c‐nu mice (RRID: IMSR_RJ: BALB‐C‐NUDE), 6–8 weeks of age and weighing 18–20 g, were purchased from Beijing Vital River Laboratory Animal Technology Co. Ltd. (Beijing, China) and used for the metastasis studies. The mice were maintained in a specific pathogen‐free (SPF) facility with unrestricted access to standard chow and drinking water. Animals were randomly assigned to the experimental groups, with the sample size for each experiment indicated in the corresponding figure legends. For assessment of systemic tumor dissemination, 1 × 10^6^ cells were injected into the lateral tail vein, while 5 × 10^5^ cells were administered through intracardiac injection, according to the experimental design. Tumor progression and dissemination were monitored at the indicated time points by bioluminescence imaging, and photon signals were analyzed using Spectrum Living Image 4.0 software (Caliper Life Sciences, Hopkinton, MA, USA; RRID: SCR_018621). Animal health and welfare were monitored throughout the experiments, and humane endpoints were implemented whenever necessary. The study was designed in accordance with the principles of replacement, reduction, and refinement (3Rs). In vitro approaches were employed whenever feasible, the number of animals was minimized while preserving sufficient statistical power, and experimental procedures were refined to limit potential discomfort. Animal experimentation and reporting were conducted with reference to the Animal Research: Reporting of In Vivo Experiments (ARRIVE) guidelines. At the predefined experimental endpoint, mice were anesthetized and euthanized, after which metastatic tissues were harvested and fixed in 4% formaldehyde. The fixed tissues were processed for paraffin embedding and sectioned at a thickness of 5 µm. Tissue sections were subsequently stained with hematoxylin and eosin (H&E) for histopathological examination.

### 5.6 Immunoprecipitation (IP) and Protein Purification

HEK293T cells (RRID: CVCL_0063; 3 × 10^7^ cells) were transfected with the indicated plasmids and harvested for protein extraction. Cell lysates were prepared in NP‐40‐containing lysis buffer supplemented with a protease inhibitor cocktail (Roche, Basel, Switzerland). For affinity capture, lysates were incubated overnight at 4°C with Flag‐ or HA‐conjugated agarose beads (Sigma–Aldrich, Burlington, MA, USA). For recombinant Flag‐METTL3 purification, the affinity beads were extensively washed five times with wash buffer containing 300 mM NaCl, 10 mM HEPES (pH 7.4), and 0.1% NP‐40. Flag‐METTL3 was subsequently isolated using Flag magnetic beads and released from the beads by incubation with 3× Flag peptide, followed by incubation with HA‐PRAME. The resulting protein complexes were washed five times with immunoprecipitation wash buffer containing 150 mM NaCl, 10 mM HEPES (pH 7.4), and 0.1% NP‐40. The recovered proteins were then denatured, resolved by SDS–polyacrylamide gel electrophoresis, and analyzed by WB.

### 5.7 GST Pull‐Down Assay

Briefly, the coding sequences of PRAME were cloned into the pGEX‐4T‐3 vector (GE Healthcare) to generate GST‐tagged fusion constructs. Recombinant GST or GST‐fusion proteins were transformed into *Escherichia coli* BL21 (DE3) competent cells and induced with 0.5 mM IPTG at 18°C overnight. Bacterial pellets were harvested and lysed in ice‐cold lysis buffer containing 20 mM Tris‐HCl (pH 7.5), 300 mM NaCl, 1 mM DTT, 0.5% NP‐40, and protease inhibitor cocktail. GST‐fusion proteins were subsequently purified using glutathione‐Sepharose 4B beads (GE Healthcare) according to the manufacturer's instructions. For in vitro binding assays, purified Flag‐tagged METTL3 proteins were incubated with GST or GST‐fusion protein‐bound beads in binding buffer containing 20 mM HEPES (pH 7.5), 300 mM NaCl, 1 mM DTT, 0.5% NP‐40, and protease inhibitors at 4°C for 4–6 h with gentle rotation. After incubation, the beads were washed extensively with binding buffer to remove nonspecific interactions. Bound proteins were eluted by boiling in SDS loading buffer and analyzed by SDS–PAGE followed by immunoblotting using the indicated antibodies. GST alone was included as a negative control to exclude nonspecific binding.

### 5.8 Immunohistochemistry Assays (IHC)

Immunohistochemical analysis was performed on paraffin‐embedded tissue sections using primary antibodies targeting METTL3 (Huaan Biotechnology, Hangzhou, China; 1:100), PRAME (ZenBio, Chengdu, China; 1:50), TGFB2 (CUSABIO, Shanghai, China; 1:50), and p‐SMAD3 (Cell Signaling Technology [CST], Darmstadt, Germany; 1:100). Staining results were independently evaluated by two observers based on the proportion of immunoreactive tumor cells and the corresponding staining intensity. The proportion of positive cells was scored on a five‐level scale: 0 for no positive cells, 1 for < 5%, 2 for 5%–25%, 3 for 25%–50%, and 4 for > 50% positive tumor cells. Staining intensity was graded from 0 to 3, corresponding to absent, weak (light yellow), moderate (yellow‐brown), and strong (brown) staining, respectively. For each specimen, the staining index (SI) was calculated as the product of the percentage score and intensity score, resulting in SI values ranging from 0 to 12, with possible scores of 0, 1, 2, 3, 4, 6, 8, 9, and 12. The cutoff value for survival‐based classification was selected according to the heterogeneity of overall survival determined by the log‐rank test. Samples with an SI ≥ 4 were categorized as having high expression, whereas those with an SI ≤ 3 were considered to have low expression. The chi‐square (*χ* ^2^) test was used to determine the statistical associations between categorical variables.

### 5.9 Cell Invasion and Migration Assays

For Transwell‐based migration and invasion assays, 3 × 10^4^ indicated cancer cells were seeded into the upper compartments of Transwell inserts (Corning Costar Corp., Cambridge, MA, USA), either without coating for migration assays or precoated with Matrigel (BD Biosciences, San Jose, CA, USA) for invasion assays. Cells were maintained at 37°C for 22 h to allow migration or invasion through the membrane. Following incubation, non‐migrated cells remaining on the upper surface were gently removed using cotton swabs. Cells that had traversed the membrane were subsequently fixed in methanol/acetic acid (3:1, v/v), stained with crystal violet, and imaged under a microscope. For quantification, cells were counted in five randomly selected microscopic fields for each insert.

### 5.10 Luciferase Signal Detection

Cells were seeded in triplicate into 24‐well plates and cultured for 24 h before transfection. Each well was transfected with 200 ng of the indicated signaling luciferase reporter plasmid (OriGene, Rockville, MD, USA) together with 5 ng of the pRL‐TK Renilla luciferase control plasmid (Promega, Madison, WI, USA), using Lipofectamine 3000 (Invitrogen, Carlsbad, CA, USA) as the transfection reagent. After 48 h, luciferase activities were measured using the Dual‐Luciferase Reporter Assay Kit (Promega, Madison, WI, USA) following the manufacturer's instructions. Firefly luciferase activity was normalized to the corresponding Renilla luciferase activity for each sample.

### 5.11 Western Blotting (WB) Analysis

WB analysis was performed using the anti‐METTL3, anti‐E‐cadherin, and anti‐Vimentin (Huaan Biotechnology, Hangzhou, China), anti‐PRAME (Proteintech, Wuhan, China), anti‐p‐SMAD3 (CST Cell Signaling Technology, Darmstadt, Germany), anti‐Ubiquitin, anti‐K48, and anti‐K63 (Millipore, Millibeau, USA), anti‐Flag, and anti‐HA antibodies (Cell Signaling, Danvers, USA), respectively. Blotted membranes were stripped and re‐blotted with anti‐p84, anti‐ß‐actin, or anti‐GAPDH antibodies (Sigma, St. Louis, MO, USA) as loading controls.

### 5.12 RNA Immunoprecipitation (RIP)

For RNA immunoprecipitation (RIP), indicated cells were subjected to crosslinking followed by cell lysis, and the resulting lysates were incubated with anti‐Flag magnetic beads or protein G beads (Sigma–Aldrich, Burlington, MA, USA). Following immunoprecipitation, both input and immunoprecipitated RNA fractions were purified using the Magna RIP RNA‐Binding Protein Immunoprecipitation Kit (Millipore, Bedford, MA, USA) according to the manufacturer's instructions. The recovered RNA was subsequently reverse‐transcribed and subjected to quantitative PCR (qPCR) for analysis of RNA enrichment.

### 5.13 Enzyme‐Linked Immunosorbent Assay (ELISA)

The levels of TGFB2 were measured using ELISA kits (FineTest, Wuhan, China) according to the manufacturer's instructions. The absorbance was measured at 450 nm using a multifunctional enzyme spectrometer (Molecular Devices, Silicon Valley, CA, USA).

### 5.14 MeRIP–qPCR

Methylated RIP coupled with quantitative PCR (MeRIP–qPCR) was performed to detect m^6^A modification on target transcripts. Briefly, total RNA was extracted from indicated LUAD cells using TRIzol reagent (Invitrogen, Carlsbad, CA, USA). Approximately 50 µg of total RNA was subject to poly(A)+ mRNA purification using oligo(dT)‐attached magnetic beads (Invitrogen). Purified mRNA was chemically fragmented into ∼100 nucleotide fragments using RNA fragmentation buffer. Fragmented RNA was incubated with anti‐m6A antibody (Synaptic Systems, Germany) in immunoprecipitation buffer (50 mM Tris‐HCl, 750 mM NaCl, and 0.5% Igepal CA‐630) supplemented with RNase inhibitor and BSA. After incubation at 4°C overnight with rotation, immune complexes were captured using protein A/G magnetic beads and extensively washed to remove nonspecific binding. m^6^A‐enriched RNA was subsequently eluted using elution buffer containing free N6‐methyladenosine (m^6^A). The immunoprecipitated RNA was purified by ethanol precipitation and reverse‐transcribed into cDNA using a PrimeScript RT reagent kit (Takara). Enrichment of m^6^A‐modified mRNAs was analyzed with qRT‐PCR.

### 5.15 RNA Sequencing and Analysis

RNA sequencing of cell or tissue samples was performed by Novogene (Beijing, China) and NovelBio (Guangzhou, China). Sequencing reads were aligned to the human reference genome (hg19) using HISAT2 (RRID: SCR_015530) under the default alignment parameters. Gene‐level read counts were subsequently obtained with HTSeq (RRID: SCR_005514), and differential expression analysis was carried out using the DESeq2 package in R (RRID: SCR_015687). Genes with an adjusted *p*‐value < 0.05 and absolute log_2_ fold change > 1 were considered significantly differentially expressed unless otherwise indicated. Volcano plot of the DEGs was produced by ggplot2 (RRID: SCR_014601). GSEA was performed using the GSEA software (RRID: SCR_003199). Normalized enrichment score (NES) and nominal *p*‐value were calculated to evaluate pathway enrichment significance. The above data have been deposited in the NCBI GEO (http://www.ncbi.nlm.nih.gov/geo/, RRID: SCR_005012), with accession numbers of GSE292191 and GSE293159.

### 5.16 Public Single Cell Sequencing Dataset and Data Analysis

Published 10 × Genomics single‐cell RNA sequencing data *GSE123902* was obtained from the Gene Expression Omnibus (GEO) dataset of the National Center for Biotechnology Information (NCBI) website (RRID: SCR_006472). Quality control and cell type annotation were performed by the original authors of GSE123902, and a Seurat object was generated by the R package Seurat (RRID: SCR_016341) for further analyses. Cells with low‐quality transcriptomes, including those with low gene counts, extremely high UMI counts, or high mitochondrial transcript percentages, were excluded during the original preprocessing procedure. Dimensionality reduction and clustering were conducted based on principal component analysis (PCA) and uniform manifold approximation and projection (UMAP). The inferCNV (RRID: SCR_021140) was used to identify malignant epithelial cells. Specifically, epithelial cells from adjacent samples were used as normal references. Copy number variations (CNVs) were inferred and used to divide cells into 6 clusters by unsupervised hierarchical clustering. For each cluster, the inferCNV score was defined as the sum of cell‐average inferred CNVs. Clusters with apparent CNVs patterns and higher inferCNV scores were identified as tumor cells. DEG analysis adopted a pseudo‐bulk strategy using DESeq2 RRID: SCR_015687), where expressions of cells within the same sample were aggregated. The DEGs of each cell type were analyzed separately.

### 5.17 Statistical Analysis

All statistical analyses were performed using the SPSS (International Business Machines Corporation, Armonk, NY, USA, RRID: SCR_002865) statistical software package. For survival analysis, animals were divided into two groups according to PRAME expression or METTL3 mutation of LUAD cells and were comparatively analyzed using Kaplan–Meier survival analysis with log‐rank test. Comparisons between two groups for statistical significance were performed with a two‐tailed Student's *t*‐test. For comparisons involving more than two groups, one‐way ANOVA followed by appropriate post hoc tests was used when applicable. Error bars represent mean ± standard deviation (SD) derived from three independent experiments. In all cases, *p*‐value < 0.05 was considered statistically significant. Statistical significance was indicated as follows: ns, not significant; *p* < 0.05; *p* < 0.01; *p* < 0.001; and *p* < 0.0001. All experiments were performed independently at least three times under similar conditions, unless described otherwise in the figure captions.

## Author Contributions

**Han Tian**: methodology. **Hongchun Du**: methodology, clinical samples. **Hongyu Guan**: methodology. **Jiamei Guo**: methodology, experiments. **Jiyong Gu**: clinical samples. **Jueheng Wu**: supervision. **Junchao Cai**: supervision. **Meiwan Chen**: analyses of sequencing data. **Mengfeng Li**: conceptualization, supervision, writing – review and editing. **Qiaorui Bai**: methodology, experiments, analyses of sequencing data. **Shuqin Dai**: methodology, clinical samples. **Shujun Liang**: methodology, experiments. **Tianyu Tao**: methodology, supervision. **Xia Yang**: methodology, experiments, writing – original draft. **Xiang Dong**: methodology, experiments. **Xiuqiao Yang**: methodology, experiments. **Xin Zhang**: methodology, clinical samples. **Xu Li**: conceptualization. **Xuemei Xu**: clinical samples. **Yi Yang**: conceptualization, project administration, writing – original draft, writing – review and editing. **Zhiyu Ye**: methodology, experiments. **Yaxuan Zhang**: Yaxuan Zhang: methodology, experiments. All authors have read and approved the final manuscript.

## Funding

This work was supported by the Key Technologies R&D Program of Guangdong Province (2023B1111030003), the National Natural Science Foundation of China (No. 82488101, 82673714, 82373036, 8260110681, 82403091, 82303613, and 82674228), Guangdong Basic and Applied Basic Research Foundation (2023A1515010378), the China Postdoctoral Science Foundation (No. 2025M782878), and the Science and Technology Development Fund, Macau SAR (File no. 0014/2023/AKP). The funders played no role in the study design, data collection, data analyses, interpretation, or writing of the manuscript.

## Ethics Statement

The study protocol was reviewed and approved by the Medical Ethics Committee of Sun Yat‐sen University. Human primary and metastatic tumor specimens were collected at Jiangmen Central Hospital (Jiangmen City, Guangdong Province, China) under the approval of its Ethics Review Committee (Approval No. [2024] 226A). The human study was conducted in accordance with the Declaration of Helsinki, and written informed consent was obtained from all participants before sample collection. Histopathological assessment and clinical staging of LUAD specimens were performed according to the most recent Tumor–Node–Metastasis (TNM) criteria of the Union for International Cancer Control (UICC). All animal experimental procedures were approved by the Institutional Animal Care and Use Committee of Sun Yat‐sen University (approval number: SYSU‐IACUC‐2022‐B1723).

## Conflicts of Interest

The authors declare no conflicts of interest.

## Supporting information




**Supporting File**: mco271066‐sup‐0001‐SuppMat.docx

**Supporting File**: mco271066‐sup‐0002‐Table S2.xlsx

**Supporting File**: mco271066‐sup‐0001‐Table S1.xlsx

## Data Availability

The datasets generated in this study have been deposited in the NCBI Gene Expression Omnibus (GEO; RRID: SCR_005012) under accession numbers GSE292191 and GSE293159. These datasets are publicly accessible through the NCBI GEO portal and are not subject to controlled‐access restrictions. No special access permission or authorization is required to access these datasets. Publicly available TCGA‐LUAD data analyzed in this study were obtained through the National Cancer Institute (NCI) Genomic Data Commons (GDC) Data Portal. The TCGA‐LUAD datasets used in this study were publicly accessible open‐access data and did not require dbGaP authorization or other controlled‐access permissions.
