# Supplementary material for: PRAME Augments K63‐Linked Ubiquitination and Stabilization of METTL3 to Promote Lung Adenocarcinoma Metastasis
Source: MedComm (2020). 2026 Oct 19;7(11):e71066. doi: 10.1002/mco2.71066 (PMC13679842; doi:10.1002/mco2.71066)
Supplement: Supplementary file 1 [file MCO2-7-e71066-s001.docx]

**PRAME Augments K63-Linked Ubiquitination and Stabilization of METTL3 to Promote Lung Adenocarcinoma Metastasis**

Xia Yang^1,#^, Xuemei Xu^2,#^, Yaxuan Zhang^2,#^, Jiamei Guo^3^, Zhiyu Ye^3^, Qiaorui Bai^3^, Xiuqiao Yang^3^, Shujun Liang^3^, Xiang Dong^3^, Xin Zhang^4^, Jiyong Gu^5^, Shuqin Dai^6^, Hongchun Du^7^, Meiwan Chen^8^, Tianyu Tao^2^, Hongyu Guan^9^, Jueheng Wu^3^, Han Tian^3^, Junchao Cai^3^, Mengfeng Li^8,2,3,*^, Xu Li^2,*^, Yi Yang^3,*^

^1^ Department of Laboratory Medicine and Institute of Precise Medicine, The First Affiliated Hospital, Sun Yat-sen University, Guangzhou, Guangdong, China

^2^ Cancer Institute, School of Basic Medical Sciences, Southern Medical University, Guangzhou, Guangdong, China.

^3^ Zhongshan School of Medicine, Sun Yat-Sen University, Guangzhou, Guangdong, China.

^4^ Clinical Experimental Center, Jiangmen Engineering Technology Research Center of Clinical Biobank and Translational Research, Jiangmen Central Hospital, Jiangmen, Guangdong, China.

^5^ Department of neurosurgery, Jiangmen Central Hospital, Jiangmen, Guangdong, China.

^6^ Sun Yat-sen University Cancer Center, Guangzhou, Guangdong, China.

^7^ Department of Pulmonary and Critical Care Medicine, The First Affiliated Hospital, Sun Yat-sen University, Guangzhou, Guangdong, China.

^8^State Key Laboratory of Mechanism and Quality of Chinese Medicine, Institute of Chinese

Medical Sciences, University of Macau, Macau SAR, China

^9^Department of Endocrinology and Diabetes Center, The First Affiliated Hospital, Sun Yat-sen

University, Guangzhou, China.

^10^State Key Laboratory of Multi-organ Injury Prevention and Treatment, Southern Medical

University, Guangzhou, Guangdong, China.

^#^ These authors contributed equally to this work.

* Corresponding author: yangyi23@mail.sysu.edu.cn (Y. Yang); lixu56@mail.sysu.edu.cn (X.Li); limf@mail.sysu.edu.cn (M. Li).
**Supplementary figures**

**Supplementary Figure 1.**

**(A** and **B)** PRAME expression across indicated datasets. **(C)** Violin plot showing PRAME expression in metastatic versus primary tumor cells from scRNAseq GSE123902. ****: *p* < 0.0001.  **(D)** PRAME expression in published microarray dataset GSE14107, comparing H2030 HMC cells with parental cells. **: *p* < 0.01. Error bars represent the mean ± SD.

**Supplementary Figure 2.**

**(A)** Cell morphology of LUAD cells with high PRAME expression. Representative images from n = 5 random microscopic fields are shown. Scale bar: 20 µm. **(B** and **C)** Correlation analysis between PRAME expression and EMT signature in patient tumor samples from the TCGA LUAD dataset. All metastatic cases (n = 185) **(B)** and cases with distant metastasis (n = 25) **(C). (D)** WB validation of epithelial (E-cadherin) and mesenchymal (Vimentin) markers in PRAME-overexpressing and PRAME-knockdown MHC cells. **(E)** Transwell invasion and Migration assays of PRAME-overexpressing and PRAME-knockdown MHC cells. Scale bar: 100 µm.

**Supplementary Figure 3.**

**(A** and **B)** Correlation analysis of metastasis and RAR signaling pathway-related signature **(A)** or NFY downstream-related signature **(B)** by GSEA based on the RNA-seq dataset comparing metastases (n=4) with primary tumors (n=4).  **(C and D)** Invasion and migration assays of

PRAME-overexpressing LUAD cells under indicated treatment. Scale bar: 100 µm.

**Supplementary Figure 4.**

**(A)** IP validation of K6-, K11-, K27-, K29-, and K33- linked ubiquitination levels in PRAME-overexpressing HEK293T cells treated with MG132. **(B)** WB validation of METTL3 levels in LUAD cells under indicated treatment. **(C)** co-IP analyses showing interactions between recombinant HA-NEDD4 and Flag-METTL3 or Flag-PRAME. **(D)** IP validation of K63- linked ubiquitination in NEDD4-overexpressing HEK293T cells with PRAME knockdown. **(E)** Sequence alignment of METTL3 protein across multiple species.

**Supplementary Figure 5.**

**(A)** WB and qPCR validation of METTL3 protein and mRNA levels in PRAME-knockdown HMC LUAD cells. **(B)** METTL3 stability and quantifications in parental or HMC A549 cells treated with CHX for the indicated time points. **(C)** METTL3 stability and quantification in HMC A549 cells treated with K63R mutant ubiquitin in the presence of CHX for indicated time points. **(D** and **E)** WB validation of METTL3 protein levels in METTL3 knockdown LUAD cells **(D)** and in cells overexpressing either WT or K235R mutant METTL3 **(E)**. Statistical analyses were performed using a two-tailed unpaired t-test in **(A)** and two-way ANOVA in **(B** and **C)**. ns: not significant, ***: *p*< 0.001, ****: *p*< 0.0001. Error bars represent the mean ± SD from three independent experiments.

**Supplementary Figure 6.**

**(A-C)** qPCR validation of downstream TGF-ß signaling target gene expression in indicated LUAD cells. **(D)** Luciferase reporter assay showing TGF-ß pathway activation in PRAME-overexpressing LUAD cells with NEDD4 knockdown. **(E and F)** Invasion and migration assays of indicated LUAD cells. Scale bar: 100 µm. **(G** and **H)** WB validation of EMT markers in indicated LUAD cells. **(I** and **J)** Invasion and migration assays of indicated LUAD cells. Scale bar: 100 µm. Representative images from three experiments are shown. Statistical analyses were performed using a two-tailed unpaired t-test. *: *p* < 0.05, **: *p* < 0.01, ***: *p* < 0.001, ****: *p* < 0.0001. Error bars represent the mean ± SD from three independent experiments.

**Supplementary Figure 7.**

**(A** and **B)** qPCR **(A)** and ELISA **(B)** validation of TGFB2 mRNA and secreted protein levels in PRAME-overexpressing LUAD cells with METTL3 knockdown. **(C** and **D)** qPCR **(C)** and ELISA **(D)** validation of TGFB2 mRNAand secreted protein levels in PRAME-overexpressing LUAD cells with METTL3 catalytically inactive mutant. **(E)** Luciferase reporter assay demonstrating TGF-ß pathway activation in PRAME-overexpressing LUAD cells with IGF2BP3 knockdown. **(F)** WB validation of p-SMAD3 level in PRAME-overexpressing LUAD cells with IGF2BP3 knockdown. **(G)** qPCR validation of downstream TGF-ß target gene expression in indicated LUAD cells. **(H)** Invasion and migration assays of PRAME-overexpressing LUAD cells with IGF2BP3 knockdown. Scale bar: 100 µm. Representative images from three experiments are shown. Statistical analyses were performed using a two-tailed unpaired t-test. *: *p* < 0.05, **: *p* < 0.01, ***: *p* < 0.001, ****: *p* < 0.0001. Error bars represent the mean ± SD from three independent experiments.

**Supplementary Figure 8.**

**(A)** RNA-seq analysis comparing metastatic tumors (n=4) with primary tumors (n=4). Volcano plot showing -log_10_ *p*-value versus log_2_ fold change of gene expression in each tissue (left panel), as well as changes in METTL3 mRNA levels across metastatic versus primary tumors (right panel). **(B)** Validation of METTL3 expression in tumor cells using a public scRNA-seq dataset. UMAP highlighting METTL3 expression in red (left panel) and Vertical bar indicating maximum expression levels (right panel). **(C)** qPCR validation of METTL3 mRNA levels in lung tumors 28 days after intracardiac injection of A549 cells, and in brain or bone metastases from Balb/c-nu mice (n=3 per group). **(D)** qPCR validation of METTL3 expression in HMC LUAD cell lines. Representative images from three experiments are shown. Statistical analyses were performed using a two-tailed unpaired t-test. ns: not significant. ***: *p* < 0.001, ****: *p* < 0.0001. Error bars represent means ± SD from three independent experiments.
